# Birds of three worlds: moult migration to high Arctic expands a boreal-temperate flyway to a third biome

**DOI:** 10.1186/s40462-021-00284-4

**Published:** 2021-09-15

**Authors:** Antti Piironen, Antti Paasivaara, Toni Laaksonen

**Affiliations:** 1 University of Turku, Vesilinnantie 5, 20500 Turku, Finland; 2 Natural Resource Institute Finland, University of Oulu, P.O. Box 413, 90014 Oulu, Finland

**Keywords:** Migratory connectivity, Migration ecology, Waterfowl ecology, Adaptive management, Flyway ecology, Flyway management

## Abstract

**Background:**

Knowledge on migration patterns and flyways is a key for understanding the dynamics of migratory populations and evolution of migratory behaviour. Bird migration is usually considered to be movements between breeding and wintering areas, while less attention has been paid to other long-distance movements such as moult migration.

**Methods:**

We use high-resolution satellite-tracking data from 58 taiga bean geese *Anser fabalis fabalis* from the years 2019–2020, to study their moult migration during breeding season. We show the moulting sites, estimate the migratory connectivity between the breeding and the moulting sites, and estimate the utilization distributions during moult. We reveal migration routes and compare the length and timing of migration between moult migrants and successful breeders.

**Results:**

All satellite-tracked non-breeding and unsuccessfully breeding taiga bean geese migrated annually to the island of Novaya Zemlya in the high Arctic for wing moult, meaning that a large part of the population gathers at the moulting sites outside the breeding range annually for approximately three months. Migratory connectivity between breeding and moulting sites was very low (r_m_ =  − 0.001, 95% CI − 0.1562–0.2897), indicating that individuals from different breeding grounds mix with each other on the moulting sites. Moult migrants began fall migration later in autumn than successful breeders, and their overall annual migration distance was over twofold compared to the successful breeders.

**Conclusions:**

Regular moult migration makes the Arctic an equally relevant habitat for the taiga bean goose population as their boreal breeding and temperate wintering grounds, and links ecological communities in these biomes. Moult migration plays an important role in the movement patterns and spatio-temporal distribution of the population. Low migratory connectivity between breeding and moulting sites can potentially contribute to the gene flow within the population. Moult migration to the high Arctic exposes the population to the rapid impacts of global warming to Arctic ecosystems. Additionally, Novaya Zemlya holds radioactive contaminants from various sources, which might still pose a threat to moult migrants. Generally, these results show that moult migration may essentially contribute to the way we should consider bird migration and migratory flyways.

**Supplementary Information:**

The online version contains supplementary material available at 10.1186/s40462-021-00284-4.

## Background

Migration is a taxonomically widespread phenomenon comprising regular, seasonal movement of animals [1, 2]. Movements between several habitats allow animals to use spatially and temporally versatile resources and thus enables them to utilize areas where they cannot live over the whole annual cycle [3]. The downside is that migration exposes animals to changing environmental conditions and human actions in all the locations they utilize during the annual cycle [4]. Migratory animals also link ecological communities to each other, transferring changes in one community to another, which makes it crucial to understand the migratory patterns of the populations [5].

Bird migration has typically been considered a movement between “two worlds” i.e. between breeding and non-breeding (wintering) areas, with some staging sites *en route* [4]. However, this view may be complicated by moult migration, a phenomenon where a part of the population disperses (usually) outside of the breeding range during breeding season for wing moult [6, 7]. While moult migration is recognized in several bird taxa, it is most widespread and best known among waterfowl *Anatidae* [6, 8]. Most ducks and geese moult and regrow all their flight feathers simultaneously during summer, which leaves them flightless for several weeks every year [9]. In many species, a part of the population leaves the breeding area to moult somewhere else. This may have important ecological and evolutionary consequences that should be known to understand migratory behaviour and population dynamics of the species, and to successfully conserve it.

To understand the evolution and occurrence of moult migration, we should understand the general components of this movement within the conceptual movement ecology framework [10]. First, we should examine the internal factors i.e. reasons for “why to move” [10]. The flightless period reduces feeding site choice and increases the risk of predation, which emphasizes the importance of the moult area choice as a reason to move. Moult migration outside breeding areas may provide (1) longer days for feeding (it often directs northwards), (2) a possibility of exploiting newly grown, nutritious vegetation, or (3) a way of avoiding predators [6]. It may also help prevent intraspecific food competition or serve some social function [6, 8]. These hypotheses have been studied on a few occasions [11, 12], but the reasons behind moult migration remain unknown. Second, moult migration typically concerns only a part of the population, which in many bird species appears to constitute mostly immature individuals. This is interesting regarding the navigation capacity (“where to move”) of moult migrants, as it is unknown how young, inexperienced birds navigate to the moulting sites far away from their natal grounds unless they can follow some experienced birds. Before we can begin to examine factors behind the evolution of moult migration, we have to know where the birds are going, how the moult migration sites are connected with the breeding sites and the flyway of a population, and how the moult migration changes the migratory patterns compared to the individuals that do not moult migrate. Surprisingly, these characteristics are rarely known. Some of these aspects are known for a handful of populations [6, 8, 13, 14], but we are not aware of populations, for which all these basic aspects of moult migration are known.

While the reasons behind moult migration remain poorly studied, its wide-reaching impacts on migration ecology and population dynamics have begun to emerge. First, a recent study revealed that moult migration links two flyway populations that have previously been considered separate populations, forming a meta-population across the flyways [14]. This highlights the previously unknown impact of moult migration on the connections and gene flow between flyway populations. Second, moult migration may shape the entire flyway concept by linking previously unknown environments (moulting sites) that can be situated in unpredictable directions from the traditional path between breeding and wintering grounds (the “two worlds”, e.g. [4, 15]). Third, moult migration may contribute substantially to the migratory connectivity of the populations, as it can change migration routes and timing of migration for a part of the population [e.g. 15]. Finally, moult migration is known to affect the demographic parameters of moult migrants and to thereby directly contribute to the dynamics and management of migratory populations [13]. To set the scene for studying the evolutionary and ecological factors behind moult migration, and to understand the impact it has on the ecology of migratory populations, it is essential to identify population-specific moult migration patterns and moulting sites.

The Western taiga bean goose *Anser fabalis fabalis* (hereafter taiga bean goose) from the Central flyway breeds in Fennoscandia and north-western Russia, and is distributed mainly in southern Sweden, Denmark and northern Germany during the non-breeding season [16, 17]. It was discovered decades ago that a part of the population disappears from the breeding grounds in early summer [18]. An earlier list of potential moulting sites included northern Fennoscandia and continental Russia, but not Novaya Zemlya [17]. A previous satellite-tracking study found that three birds migrated from Sweden to Novaya Zemlya, which suggested the possibility that moult migration to the high Arctic could take place [19]. Based on this knowledge, the latest population review on the species noted that moult migration to Russia, potentially mainly to Novaya Zemlya, occurs [16]. However, the taiga bean goose moulting sites, the extent of moult migration in the population and the impact of moult migration to the migration patterns of the population have remained unknown.

Here, we examine via satellite-tracking (1) how commonly the taiga bean geese from several different breeding areas moult migrate outside the breeding areas; (2) where they moult; and (3) whether there is connectivity between breeding and moulting areas. We further examine (4) how this behavior changes the length, timing and route of their migration compared to the individuals that do not moult migrate. Finally, we discuss the evolution of moult migration and the importance it has for the flyway concept, individual migratory behaviour, migratory populations and their conservation.

## Material and methods

### Field methods

**Fig. 1:**
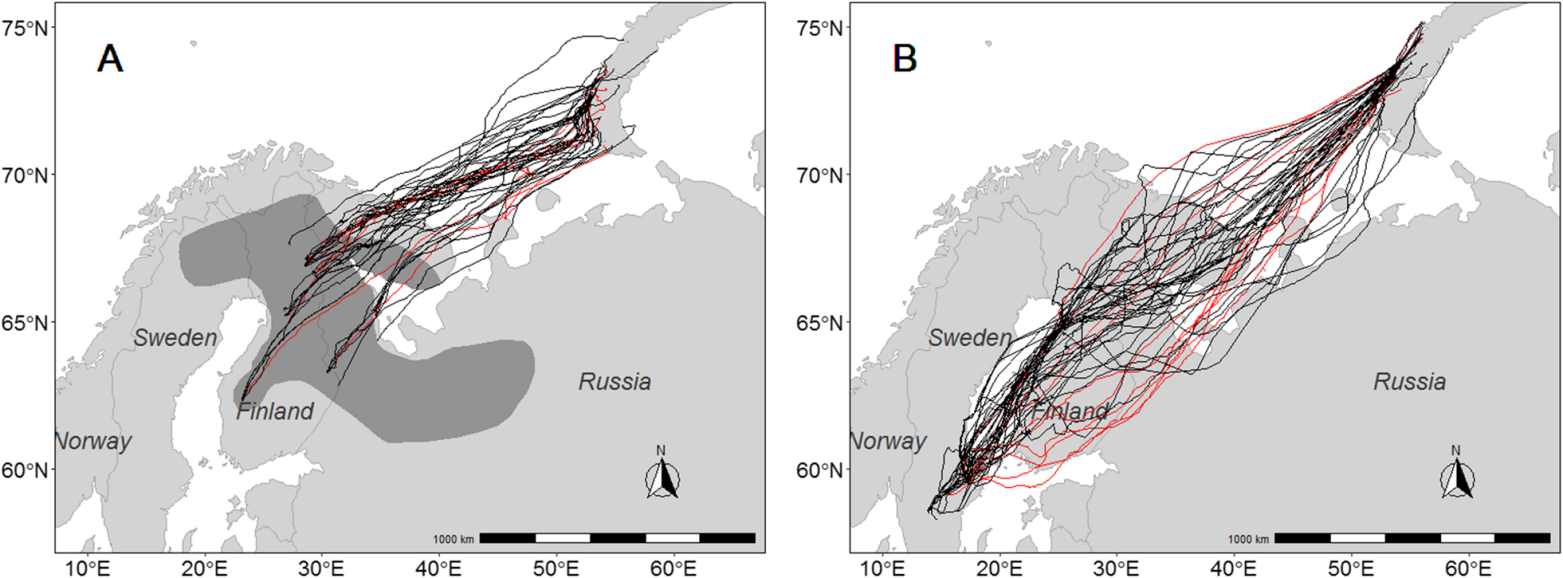
The moult migration routes of satellite-tracked taiga bean geese from Finland and Russia to Novaya Zemlya (map A) and breeding distribution of taiga bean goose in the Central Flyway (shaded area in map A). Map B represents the autumn migration routes of the same birds from Novaya Zemlya to Sweden after moult. Red lines denote routes in 2019 and black lines routes in 2020. The breeding distribution is redrawn after [16]

Taiga bean geese were caught for global positioning system (GPS) transmitter deployment during spring and summer 2019 and spring 2020 on five sites in Finland (Fig. 1). The catching sites on the Finnish breeding grounds are located at Virrat in South Ostrobothnia (latitude (lat) 62° 22′ longitude (lon) 23° 16′), Lieksa in North Karelia (lat 63° 16′ lon 30° 28′), Pudasjärvi and Utajärvi in North Ostrobothnia (lat 65° 04′ lon 26° 50′ and lat 65° 12′ lon 26° 52′, respectively), and Salla in Lapland (lat 66° 51′ lon 28° 36′). Birds breeding in Russia were caught on staging sites at Outokumpu and Liperi (lat 62° 42′ lon 29° 07′) in North Karelia. We caught birds in breeding areas using cannon-nets combined with short-term artificial feeding. Catching sites were located on small fields, mires, or at the edge of ponds and they were prepared prior to catching events by feeding geese with grain from several days up to three weeks. To mark breeding pairs on their specific breeding sites in Finland, geese were caught in pairs immediately after the first geese had arrived at the breeding grounds in spring. On staging sites at Outokumpu, birds were caught using cannon-netting on agricultural fields.

All caught birds were sexed with a cloacal examination and aged based on the shape of the wing coverts. GPS transmitters were deployed only onto birds of age + 2 cy (calendar year) (see Additional file 2: Table 1 for individual data on marked birds). We used OrniTrack-44 solar-powered GPS-GSM (global system for mobile communication) neckcollars produced by Ornitela UAB. The collar weighs appr. 45 g, which is under 3% of the weight of an adult female taiga bean goose (Piironen A., unpublished). The transmitter logs GPS positions and sends data to the server via a GSM/GPRS (general packet packet radio service) network either by e-mail or SMS (short message service).

Individuals with only limited data sets were used for analysis when data allowed. For example, data from birds that moult migrated, but did not send any data after departure, were used to calculate the beginning of moult migration even if they could not be included in other analyses. Seven individuals were tracked over both years, and their data are probably non-independent between the two years. To avoid pseudoreplication, we only used data from one year for these individuals when we merged the data from both years for analysis. Likewise, our data included two pairs, which produce non-independent data as paired individuals usually move tightly together. Therefore, we used data from one member of a pair when it was more appropriate than to use both individuals (see Additional file 3: Table 2 for sample sizes used in different analyses).

### Data and analysis

GPS resolution was set to one position per ten minutes, except for three birds in 2019, whose GPS resolution were set to one hour. Before the analyses, we excluded GPS noise from the data (i.e. locations with lat 00° 00′ lon 00° 00′). To ensure the best possible quality of the locations, we only used locations with hdop (horizontal dilution of precision of the GPS fix) values ≤ 2. We assessed the nesting status and success for females using location revisitation metrics following [20]. We identified possible nest sites from the period 15th April–30th June from revisited places with the following criteria: (1) Nest site (defined as a 60-m radius to account for small-scale movements around the nest and bias in the GPS locations [21]) must be visited in at least six consecutive days (corresponding to average clutch size and laying approximately one egg per day [22]), (2) it must be visited in at least 50% of days between first and last visit, and (3) at least 50 locations must be from the site. We note that the last two criteria are subjective, but necessary to exclude other often visited sites such as feeding and roosting sites and thereby to reduce the amount of candidate nest sites. However, we think that any true nest site should fill these criteria. If the bird’s track included at least one site filling these criteria, we considered that the bird attempted to nest in that year. Vice versa, we considered any bird that did not fill the criteria as a non-breeder. From the candidate nest sites, we selected the most visited site for each bird and each breeding season as the nest site (bean geese are not known to re-nest after unsuccessful attempts [18]). We assessed nesting success for females by comparing incubation duration to the previously known incubation duration for the species (27–29 days [22]). We considered that the bird started incubation when the daily nest site attendance was at least 70%. If the amount of consecutive incubation days was at least 28, we considered nesting successful (i.e. at least one egg hatched). We note that these quantitative assessment rules include some subjective threshold values. However, the conclusions about nesting on these bases are in accordance with what could be evaluated from it when following the tracks of individual birds from the (high resolution) satellite-tracking data.

We identified breeding males from non-breeding males when they joined the females and goslings after hatching i.e. stopped flying and started to move continuously by walking before the mid-June (moult period). We considered that a male was with brood, if at least 99 percent of daily locations indicated movement at a speed ≤ 20 km/hour (km/h) (i.e. the distance covered between two locations indicated movement at a speed ≤ 20 km/h). We note that this assumption carries a risk of a misjudgement. According to our observations, taiga bean goose males usually do not indicate nest location with their movements (the male does not visit the nest often or guard it intensively). Thereby, a breeding male may be judged to be a non-breeder if the nest is lost before hatching.

We assessed brood rearing success for both males and females using the same criteria (bird is with a brood if at least 99% of daily locations indicated movement at a speed ≤ 20 km/h). We judged that a brood was lost when parents with a brood suddenly began flying after a non-flight period following hatching and before moult or moult migration. Individuals caught and GPS-tagged during moult or right after moult were in flocks including both adults and juveniles, and we thereby considered all these adults to be successful breeders.

The natal origin of non-breeding birds often remains unclear, and it is therefore uncertain where spring migration ends and moult migration begins. This was particularly the case with four birds (two pairs) marked at staging areas in North Karelia in 2020. They flew to the Kola Peninsula on 4th May, staged there for over a month, and moult migrated to Novaya Zemlya on 14th June. Due to a very long staging period in the Kola Peninsula, we considered it more likely that these birds originated from Russia rather than Finland, and their moult migration is thus considered to begin from the Kola Peninsula instead of Finland.

We performed a phenology analysis separately for years 2019 and 2020 whenever the nature of the event suggested considerable variation between years. We considered the moulting period for each bird to be the longest period during which its speed at locations did not exceed 20 km/h or the distance between two points did not require a speed of ≥ 20 km/h. With these criteria, moulting period length obtains biologically reasonable values, although variation is probably larger than the true variation in moulting period length. This is probably due to inaccuracy in speed sensor values and location precision (too high speed during the moulting period resulting in overly short moulting periods) or to a time lag between re-gaining the ability to fly and recording the first flight observation (resulting in excessively long moulting periods).

Low GPS resolution increases the uncertainty of bird movements between locations, which leads to larger estimates for utilization distributions compared to individuals with short location intervals. Increasing location intervals also decrease the ability to accurately determine the moult period. For accurate and comparable estimates for moult timing and utilization distributions during moult, three birds with location intervals of 1 h in 2019 were removed from these analyses. Utilization distributions during moult were estimated using dynamic Brownian Bridge Movement Models [23] with a window size of 29 locations and a margin size of 11 locations. We estimated the strength of migratory connectivity between breeding and moulting locations by calculating Mantel’s correlation (r_M_, correlation between two matrices) with 1000 bootstrap runs for distances between individuals on breeding grounds and moulting sites. Mantel’s test is commonly used to calculate correlation between two matrices and in this case, we used it to test whether the birds breeding in separate areas also moult in separate areas. We tested the effect of moult migration to the timing of autumn migration by fitting a linear model with the log-transformed arrival time in Sweden as a response variable and year and moult migration status (moult migrated or stayed at the breeding grounds) as categorical explanatory variables. All analyses were performed using packages Rnest [20], move and MigConnectivity [24] and related packages in R software version 4.0.3 [25].

## Results

### Moult migration routes and timing of moult migration

All satellite-tracked non-breeders (7 individuals in 2019 and 28 in 2020) and failed breeders from Finland and Russia moult migrated to Novaya Zemlya for wing moult (4 individuals in 2019 and 13 in 2020). Four out of ten (40%) and six out of 20 (30%) birds that started breeding were successful in breeding in 2019 and 2020, respectively. We note that stress caused by capturing and marking (especially close to breeding season) might negatively affect the breeding success of the birds. Thereby, these breeding successes should be treated as minimums in years 2019–2020. Four birds that bred in 2019 skipped breeding in 2020 and were considered non-breeders. Altogether, the majority of birds alive thus moult migrated in these two years, as we documented 10 successful breeding events, 17 failed breeding events and 35 non-breeding events, of which the latter two always lead to moult migration.

On average, non-breeders began their moult migrations on 8th June ± 3.4 (s. d.) days in 2019 (n = 7) and 14th June ± 5.1 days in 2020 (n = 26). Failed breeders began moult migration on average 18.8 ± 7.2 days after losing the nest or brood (n = 16), which means 24th June ± 14.9 and 8th June ± 8.6 days in 2019 and 2020, respectively.

**Fig. 2:**
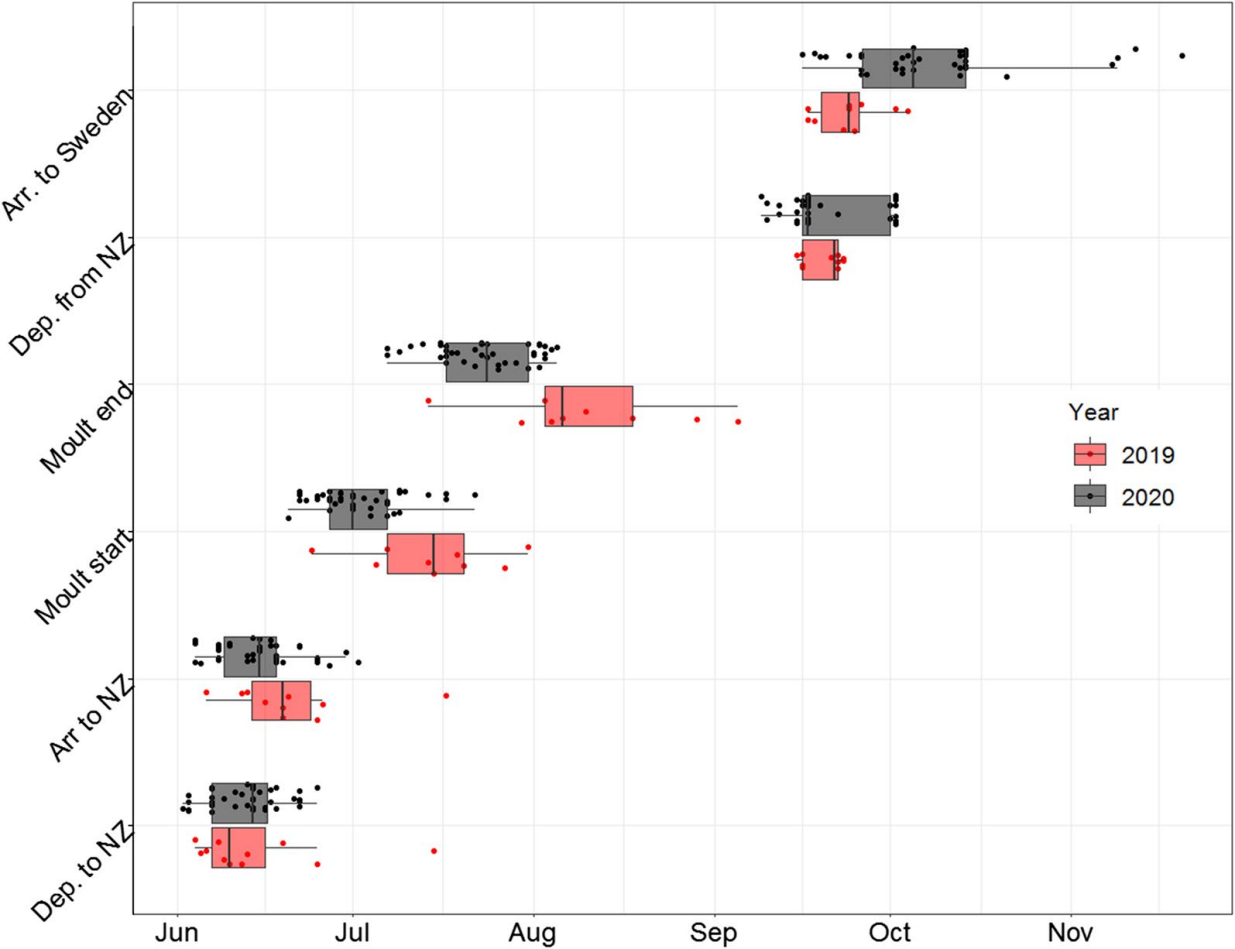
Timing of moult migration, moult, and autumn migration of satellite-tracked taiga bean geese moulting in Novaya Zemlya in 2019–2020. Boxes represent the interquartile range (IQR), vertical bars the median, and the whiskers the 25th percentile − 1.5*IQR and 75th percentile + 1.5*IQR

Moult migrants from Finland and Russia flew straight to southern Novaya Zemlya (Fig. 1). Most individuals flew almost continuously (stopped for less than one day) from the breeding sites to Novaya Zemlya, while some birds staged shortly (5.5 ± 4.9 days) on the way. The birds arrived at Novaya Zemlya on 20th June ± 11.1 days in 2019 (n = 10) and 15th June ± 7.2 days in 2020 (n = 39, see Fig. 2). After arriving on the island, most birds headed straight to the moulting sites in the central parts of Novaya Zemlya, whereas some individuals staged shortly before reaching the moulting sites. Moult began 18.69 ± 8.7 days after arrival at Novaya Zemlya (n = 42).

### Moulting sites, timing of moult and connectivity between breeding and moulting sites

Moulting began 13th July ± 11.1 days and ended 8th August ± 15.4 in 2019 (n = 10). In 2020, moulting began 2nd July ± 7.7 days and ended 23rd July ± 8.1 (n = 41). In merged data from both years, moulting began 5th July ± 9.8 days and ended 27th July ± 12.3 days (n = 44). Thereby, the moult period took on average 21 days (Fig. 2).

**Fig. 3:**
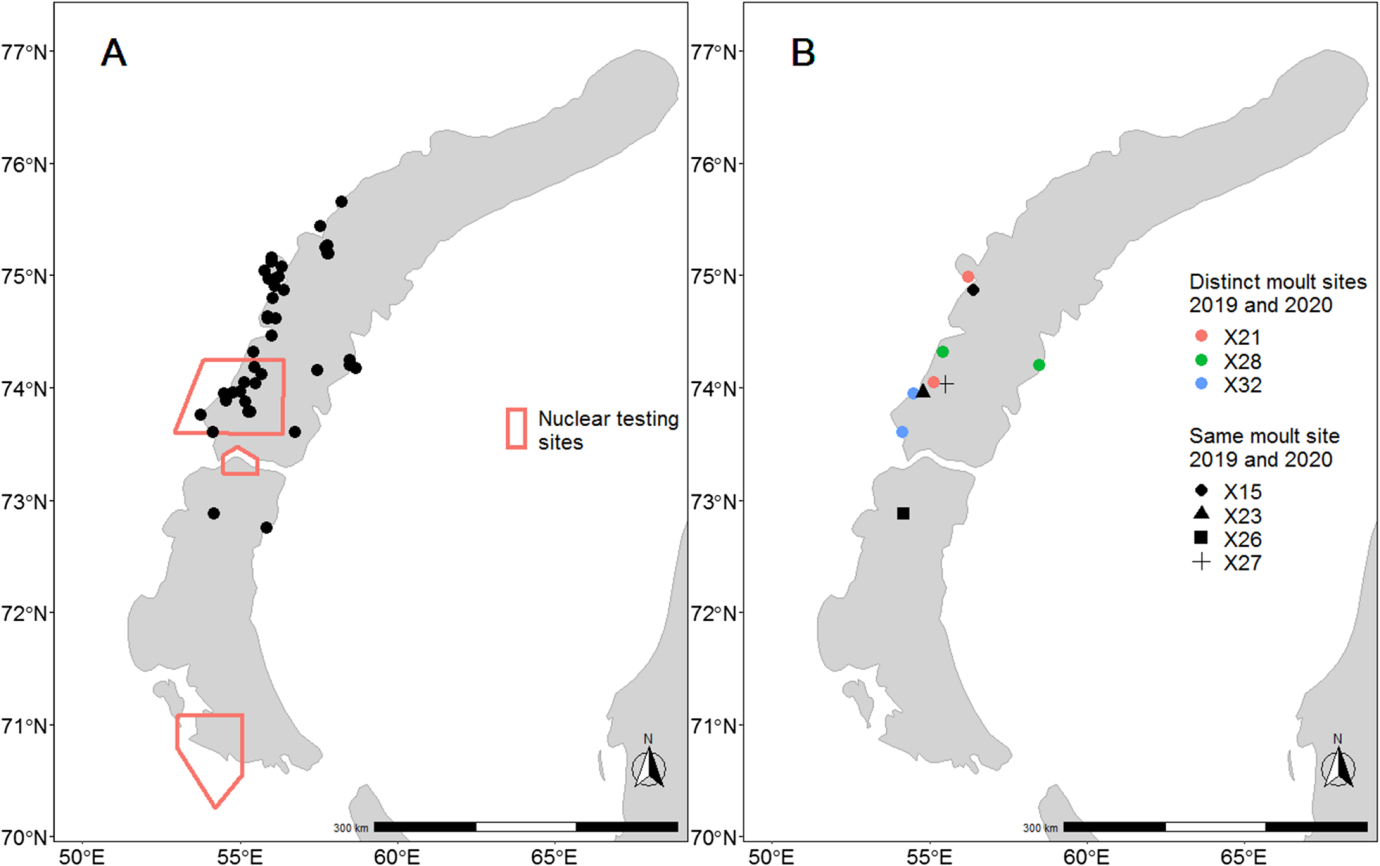
Moulting site locations of satellite-tracked taiga bean geese in 2019–2020. Map A shows all recorded moulting sites and former nuclear testing sites. Map B shows the moulting locations of individuals tracked to Novaya Zemlya over both years. Nuclear testing sites are redrawn after [25]

**Fig. 4:**
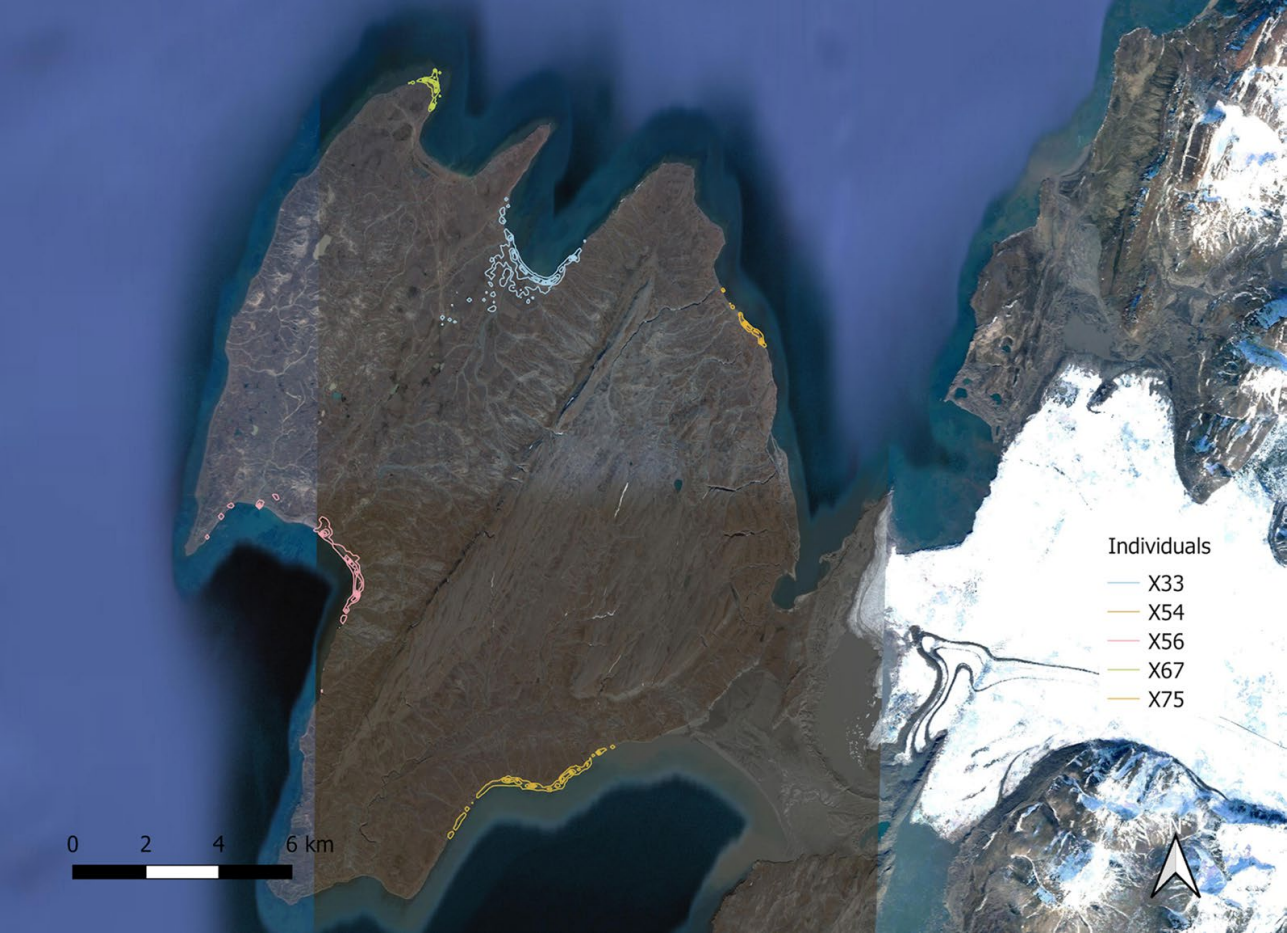
Estimated utilization distributions of five taiga bean geese during moult at Novaya Zemlya in 2020. The outermost contour is a 0.95 probability contour for each individual. Background map: Google© 2021 Terrametrics, Maxar Technologies

The taiga bean goose moulting sites in Novaya Zemlya are located in the middle part of the island, between 72° and 76° latitudes (Fig. 3). Many of the birds were moulting in the area of the most active nuclear testing in the archipelago (Fig. 2). Seven individuals were tracked to Novaya Zemlya in both years of the study. Four of these birds moulted on the same site in both years, whereas three individuals changed moulting sites between years (Fig. 3). All birds spent their moult periods close to water on the seacoast, on tundra lakes, or in river valleys. Examples of utilization distributions during moult are shown in Fig. 4 and utilization distributions for all individuals are in the Additional file 1.

**Fig. 5:**
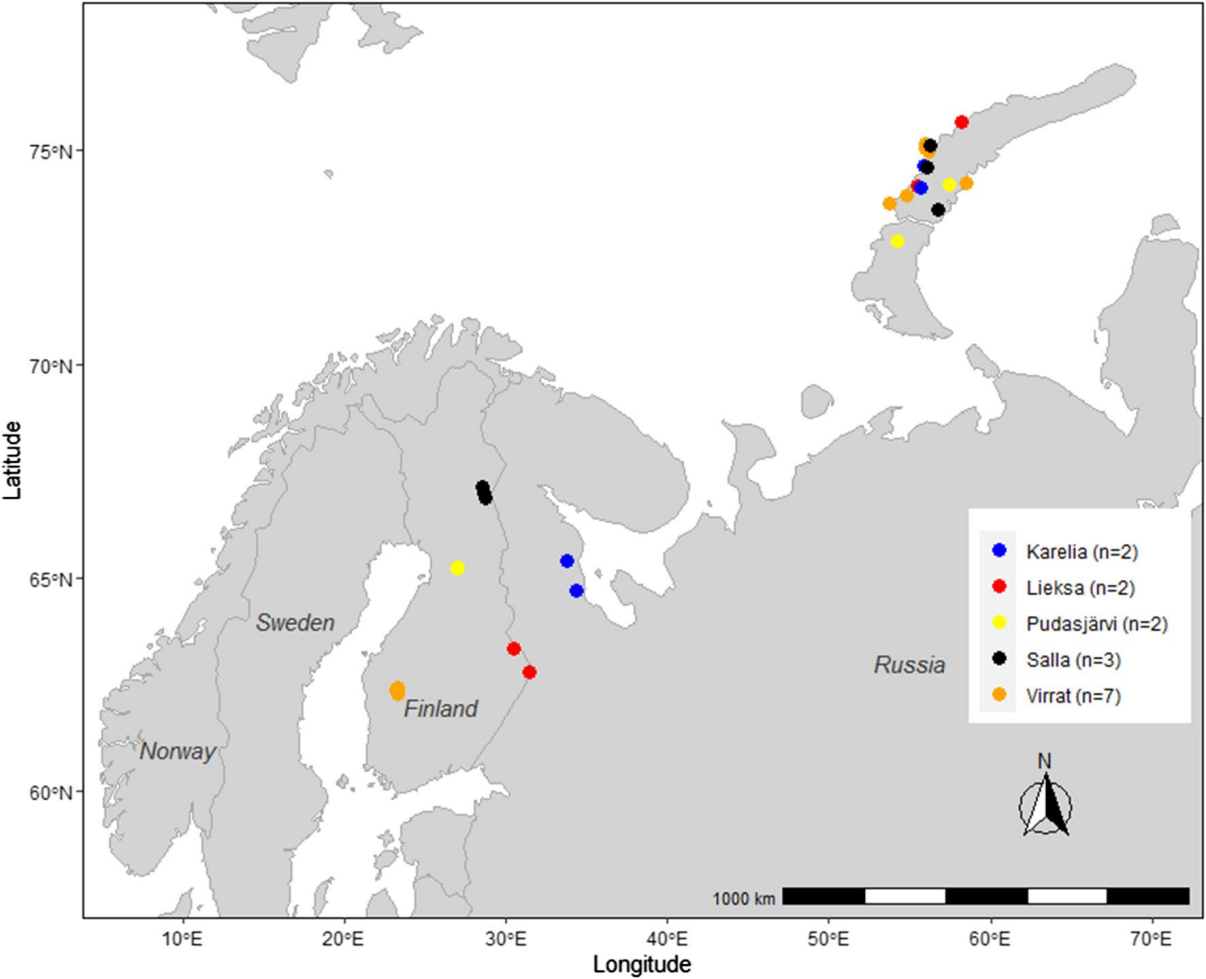
Connectivity between breeding sites and moulting sites in Novaya Zemlya. Birds breeding in the same area are denoted with the same colour. Mean estimate for Mantel’s correlation r_M_ for distances between individuals on breeding and moulting sites is − 0.001 (95% CI − 0.1562–0.2897)

We estimated connectivity between breeding sites and moulting sites in Novaya Zemlya only for failed breeders, as the non-breeders obviously lacked a breeding area. Mean estimate for Mantel’s correlation r_m_ with 1000 bootstrap samples for distances between individuals on breeding and moulting sites was − 0.001 (95% CI − 0.1562–0.2897), indicating low connectivity between breeding and moulting sites (Fig. 5).

### Autumn migration

We define the onset of autumn migration here as the time when birds leave from Novaya Zemlya, but we note that some birds had small-scale movements within the island before leaving it. As these movements can appear in any direction (and be back and forth movements), they are not considered to be part of autumn migration here. The satellite-tracked taiga bean geese left Novaya Zemlya in late September or early October. Average departure date was 19th September in 2019 (± 3.4 days; n = 10) and 20th September in 2020 (± 7.7 days; n = 39). Average time lag between the end of moult and departure was 41.8. ± 16.6 days in 2019 (n = 10) and 58.9 ± 11.1 days in 2020 (n = 39). Six out of ten birds (60%) and 15 out of 41 birds (37%) stayed on their moulting sites until leaving the island in 2019 and 2020, respectively. The rest of the birds had small-scale movements within the island before crossing the Barents Sea.

Migration routes from Novaya Zemlya to the staging areas in Sweden are presented in Fig. 1b. The migration corridor was wide, reaching from the south coast of the White Sea to the northwest corner of the Kola Peninsula. The main route followed the west coast of Finland and crossed the Baltic Sea north of Åland islands. During autumn migration, the birds that staged for at least one day did so only in Finland (not in the Kola peninsula or elsewhere in Russia). In 2020, 24 birds (62%, n = 39) staged in Finland, and the duration varied between 1 and 54 days (median 21 days, n = 30). In 2019, only two birds (20%, n = 10) stopped in Finland during autumn migration, with duration times of 7 and 17 days.

**Fig. 6:**
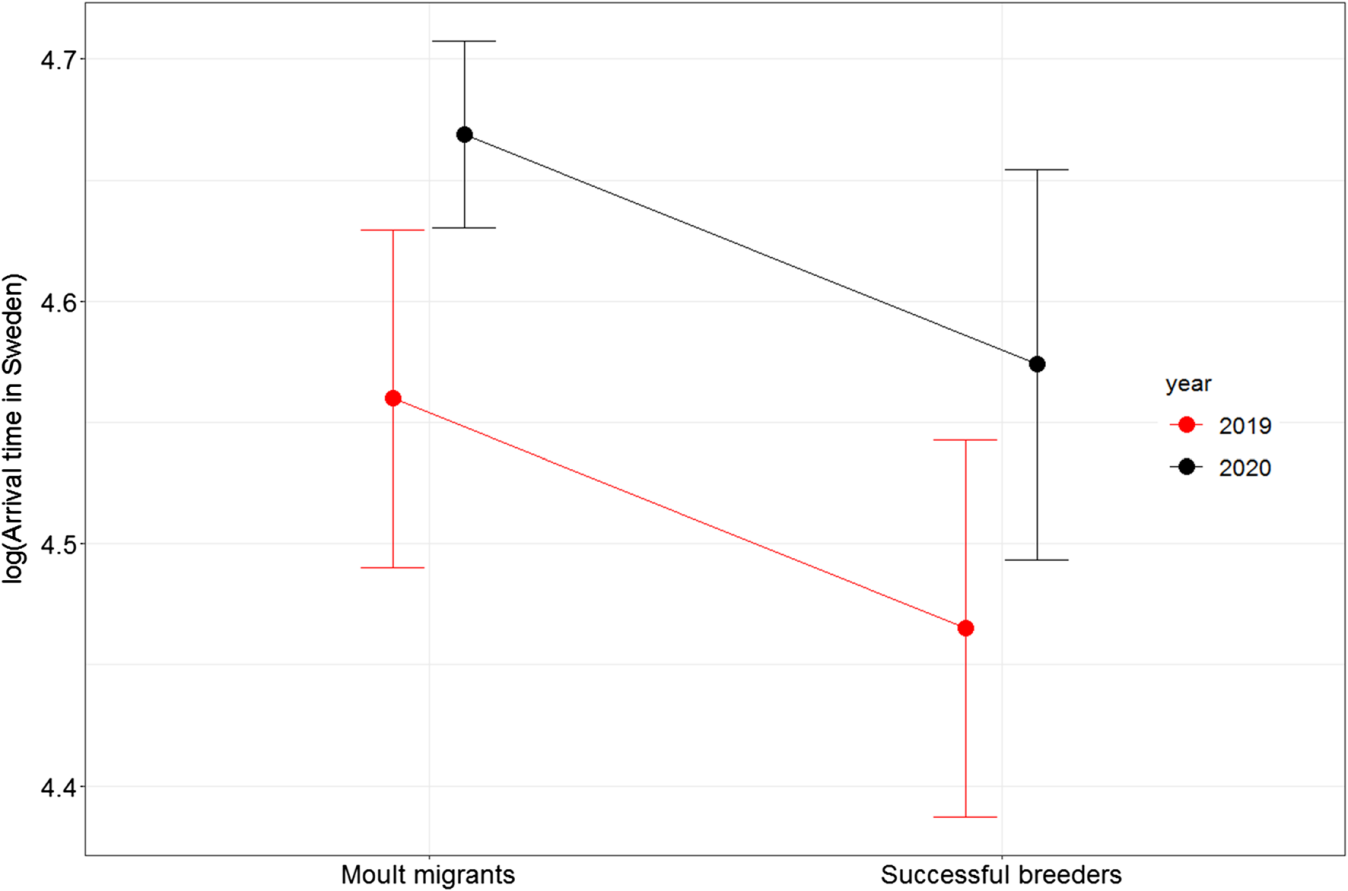
Impact of moult migration to the timing of taiga bean geese autumn migration. Figure shows model predictions with 95% confidence intervals for moult migrant and successfully breeding (moulting at breeding grounds) in years 2019–2020

The main destinations in Sweden were located in Enköping and Örebro, where the birds arrived from late September to mid-November. Mean arrival date was 24th September ± 5.8 days (n = 10) in 2019 and 8th October ± 15.3 days in 2020 (n = 38). In comparison, successfully breeding birds from Finland arrived in Sweden on 19th September ± 6.9 days (n = 7) and 25th September ± 18.2 days (n = 6) in 2019 and 2020, respectively (note that sample size of successful breeders in 2019 increased from four to seven because three additional birds were tagged while rearing broods). Despite the fact that both groups migrated later in 2020 than in 2019 (year: t_1,60_ = 2.91, *p* = 0.005), moult migrants arrive in Sweden earlier in autumn than successful breeders (migratory status: t_1,60_ =  − 2.31, *p* = 0.024, Fig. 6).

Moult migration increased the individual annual migration distance (compared to migration only between breeding and wintering grounds) by 6140 ± 758 km (n = 51). The overall annual migration distance was 2.18 times longer for moult migrants than successful breeders moulting at the breeding grounds (Additional file 4: Table 3).

## Discussion

Our results show that both non-breeding and unsuccessfully breeding taiga bean geese migrate from their boreal breeding grounds to moult on Novaya Zemlya in the Arctic Ocean. This means that a large proportion of the Central Flyway taiga bean goose population is concentrated in this relatively small area every year. Bean geese spend approximately three months on the island, around four times longer than the typical duration of moult. This shows that the high Arctic is as relevant an environment for them as the boreal breeding and temperate wintering areas. The observed moult migration changes migratory performance of the population by increasing the length of the annual migration distance and delaying the autumn migration for a non-random part of the population. An alarming finding is that the moulting area is close to the most active historical nuclear testing sites in the world in a biome that is facing rapid climate change. Altogether, the inclusion of the high Arctic to the migration system that has mainly been considered boreal-temperate links the three ecological communities to each other, raises interesting questions on the potential evolution of the flyway and has important implications for population censuses and management.

### Impacts of moult migration on individual behaviour

The observed moult migration behaviour means that breeding success and breeding status have a major impact on individual migratory behaviour of the taiga bean geese. By spending the summer in Novaya Zemlya, moult migrants more than double the length of their annual migration route and delay their autumn migration in comparison to successful breeders. Migratory connectivity between breeding and moulting sites have not been previously studied as a previously unknown portion of the population participates in moult migrations, and the specific moulting sites on Novaya Zemlya, or other destinations, have been generally unknown. As shown in Fig. 5, migratory connectivity between breeding and moulting sites in Novaya Zemlya is very low, meaning that individuals from different origins mix with each other during the moulting season. Although the pair formation in goose populations is traditionally thought to take place during winter, it has recently been shown that individuals from two populations of greater white-fronted geese *Anser albifrons* changed their flyway (population) in shared moulting sites, probably through pair formation during moulting [14]. In a similar manner, it is possible that low connectivity between breeding and moulting sites could contribute to the gene flow inside the Central Flyway taiga bean goose population, if at least some pair formation takes place during the moulting. However, this should be investigated in future studies. In general, more attention should be paid to migratory connectivity between breeding and moulting sites, and its consequences to the gene flow inside and between moult migrating populations.

### Population consequences of moult migration

All satellite-tracked non-breeding and unsuccessfully breeding taiga bean geese in this study (from Finland and Russia) and a previous study (from Sweden [19]) have moult migrated to Novaya Zemlya. This indicates that a large part of the population is there in the late summer, which was previously unknown. This can be concluded because the bean geese typically only start breeding in their third year (the largest cohorts being non-breeders) and a large proportion of breeding-age individuals fail to breed successfully. That clearly a minority of adult birds are successful breeders can be seen in the counts of birds in Sweden, in which the proportion of first-year birds has been 7.7–14.2% of the whole population [16]. With a conservative average brood size estimate of two individuals (each successful pair having two offspring), this would mean that 15.2–28.4% of the population are successful breeders and their offspring, while all the rest are non-breeders and unsuccessful breeders. In concert with this, we found that 40 and 29% of the breeding attempts of the tagged birds were successful in 2019 and 2020, respectively. Late snowmelt in northern Finland in 2020 probably contributed to low breeding success, but also to the fact that four individuals which bred in 2019, skipped breeding in 2020. However, our results show that moult migration can strongly contribute to the spatio-temporal distribution of the population and that it can be an integral part of the migratory flyway of a population. Therefore, more attention should be paid to moult migration in future research on migratory birds with synchronous wing moult and potential moult migration (see [8] for relevant taxonomic groups).

### Evolution of the moult migration behaviour

The extensive moult migration far outside of the breeding range raises two interesting questions regarding the evolution of the behaviour: How does it develop in individuals and which selective factors are behind it? The evolutionary history behind the moult migration is interesting regarding the question on how the birds navigate to the moulting sites (“where to move” [10]), as the history might reveal whether the navigation to the island is more likely to be based on genetics or social learning. Currently, we can only speculate about the evolutionary history of the behaviour, and we are not aware of any other populations for which it would be known either. This would be an important topic for future studies, to better understand the development of current flyway structures. However, that a large part of the population is now known to undertake moult migration would in this case seem to suggest strong selective benefit for doing so. The selective factors behind moult migration should be studied to understand the birds internal factors for this movement (“why to move”). Avoidance of predators, food supply or temperature (niche tracking) are plausible candidates, but whether and which of these factors play a role remains to be examined. This is also relevant because all of these factors may be changing, which may change the scene of selection for moult migration. It is apparent that moult migration to the high Arctic includes at least energetic costs of flying for moult migrating individuals, which must be outweighed by one or several fitness benefits. To understand the evolution of moult migration and the flyways related to moult migrating populations, it would thus be essential to reveal the fitness gains for individuals caused by moult migration.

### Conservation concerns of moulting in a high Arctic nuclear testing site

Novaya Zemlya has been one of the most active nuclear testing sites in the world [26], and the taiga bean goose moulting sites are located in the close proximity of the testing areas (Fig. 3). Besides nuclear tests, various types of nuclear waste have been buried in soil and shores of Novaya Zemlya [27, 28]. Data on soil radioactive contamination in Novaya Zemlya is scarce [29], but contaminated areas are reported at least near nuclear waste dumping sites [28]. A large part of the taiga bean goose population thus gathers annually to an area that has exposed the population to the direct effects of detonations, nuclear fallouts and leaks of nuclear waste. The extent of which the taiga bean goose population has been exposed and is currently exposed to radiation is unknown, but several dozen cohorts have at least fed on radioactive-contaminated food in Novaya Zemlya over many years. While the potential historical impacts of the radioactive exposure on the population are unknown, they have clearly been possible, and the potential current and future exposure should be investigated.

Additionally, moult migration to the high Arctic exposes the taiga bean goose population also to the rapid impacts of climate change to the arctic ecosystem [30]. The frequency of extreme weather conditions increase due to global warming, along with impacts on arctic vegetation, such as shrub expansion [31]. Regular gathering of the taiga bean geese annually in a small area in the high Arctic thus makes the population vulnerable to extreme weather conditions [32] and to unfavourable changes in vegetation.

### Research and management implications

Moult migration to Novaya Zemlya has direct impacts on the on-going population monitoring activities in the Central Flyway [33]. Monitoring of the taiga bean goose breeding population on the breeding grounds during the moult period has recently been developed in Finland (Paasivaara & Laaksonen, work in progress). Our results show that this monitoring scheme needs to take into account that breeding success has a strong impact on taiga bean geese numbers on the breeding grounds during moult. If the goal of these counts is to monitor the size or development of the breeding population, breeding success must be carefully monitored to separate yearly changes in breeding population size from the yearly changes in breeding success. Second, the productivity of the Central Flyway population is estimated by counting juvenile ratios in autumn flocks in Sweden [16]. Moult migrants returning from Novaya Zemlya consist exclusively of non-juvenile birds that arrive in Sweden from late September to mid-November, thereby decreasing the juvenile ratio observed in Sweden during autumn. If counts are carried out early in autumn, there is a risk of overestimating the juvenile ratio (productivity), as all sub-adults and a large proportion of the breeding adults may still be in Novaya Zemlya. Additionally, our results indicate that the arrival date of moult migrants to Sweden can have substantial variation between years.

## Conclusions

Our results show that moult migration can have a major impact on migratory behaviour of birds, linking both breeding status and success to individual migratory performance and spatio-temporal occurrence of the populations. It can create unexpected connections between ecological communities in different biomes, such as the connection between boreal forests and high Arctic presented in this study. Revealing these connections and examining their consequences to both moult migratory populations and the ecological communities connected by the moult migrants are exciting questions for future research. As shown in this and other studies, moult migration can also expose populations to several anthropogenic pressures, potentially decreasing the survival of individuals [13]. On the other hand, factors behind the evolution of moult migration are rarely studied. To understand the evolution of moult migration and its current impact on bird populations, the impact of moult migration to survival rates and future breeding success would be essential to investigate in future studies.

## Supplementary Information

**Additional file 1**. Utilization distributions in Novaya Zemlya during moult for all birds tracked in this study.

**Additional file 2. Table 1**. Individual data on taiga bean geese marked with GPS-transmitters during 2019–2020.

**Additional file 3. Table 2**. Length of migration routes, duration of migration and yearly additional journey caused by moult migration.

**Additional file 4. Table 3.** Explanation of sample sizes used in the analysis.

## Data Availability

The datasets generated and analyzed during the current study are available in the Movebank Data Repository, https://doi.org/10.5441/001/1.22kk5126 (Piironen et al., 2021). Sensitive data (locations showing nest locations) are excluded from the publicly available data.

## Abbreviations

GPS: Global positioning system
GSM: Global system for mobile communications
GPRS: General packet radio service
SMS: Short message service
km/h: Kilometres/hour
lat: Latitude
lon: Longitude
