# Supplementary material for: Birds of three worlds: moult migration to high Arctic expands a boreal-temperate flyway to a third biome
Source: Mov Ecol. 2021 Sep 15;9:47. doi: 10.1186/s40462-021-00284-4 (PMC8444479; doi:10.1186/s40462-021-00284-4)
Supplement: Supplementary file 1 — Additional file 1. Utilization distributions in Novaya Zemlya during moult for all birds tracked in this study. [file 40462_2021_284_MOESM1_ESM.pdf]

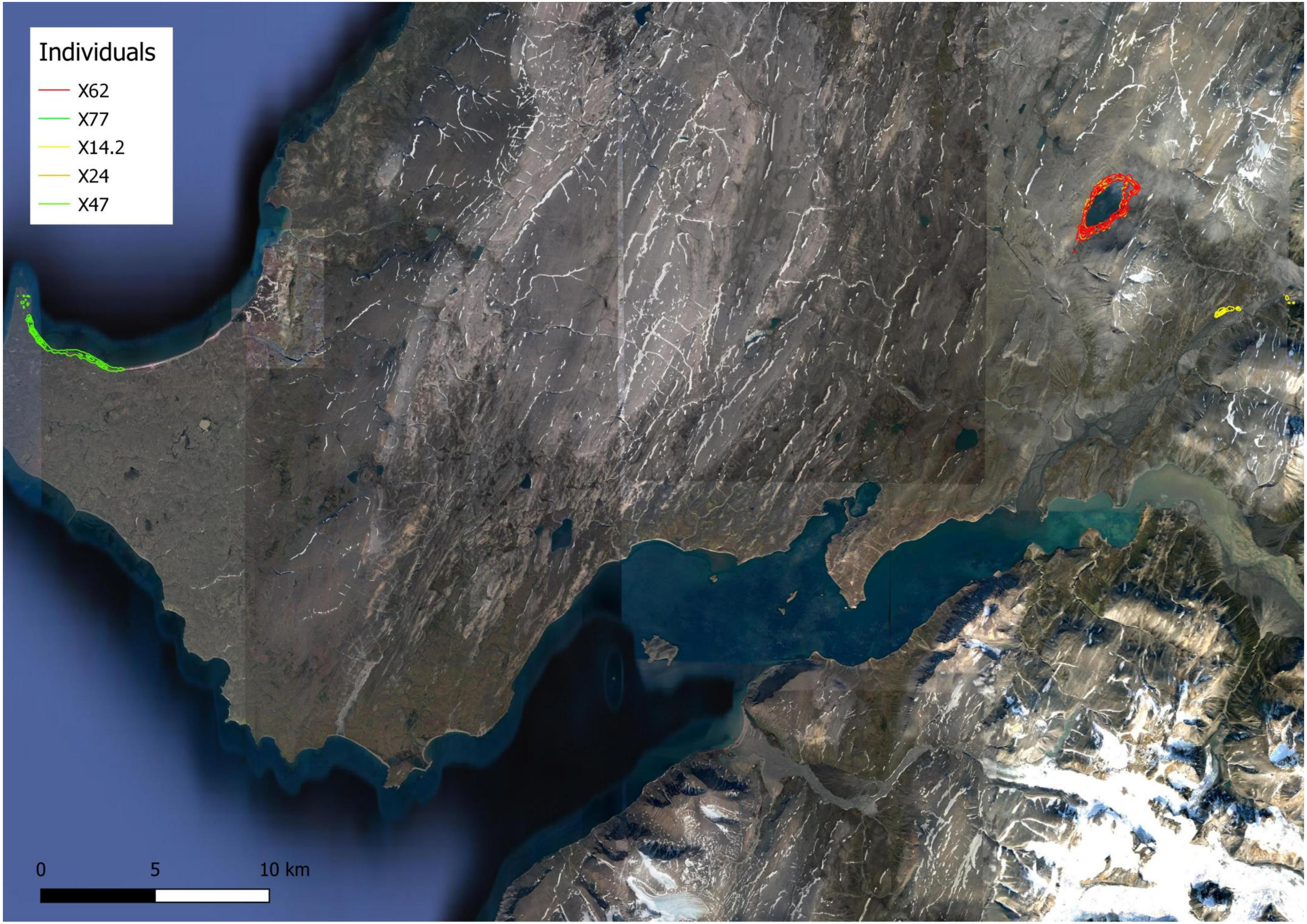
Individuals
X62
X77
X14.2
X24
X47
0
5
10 km

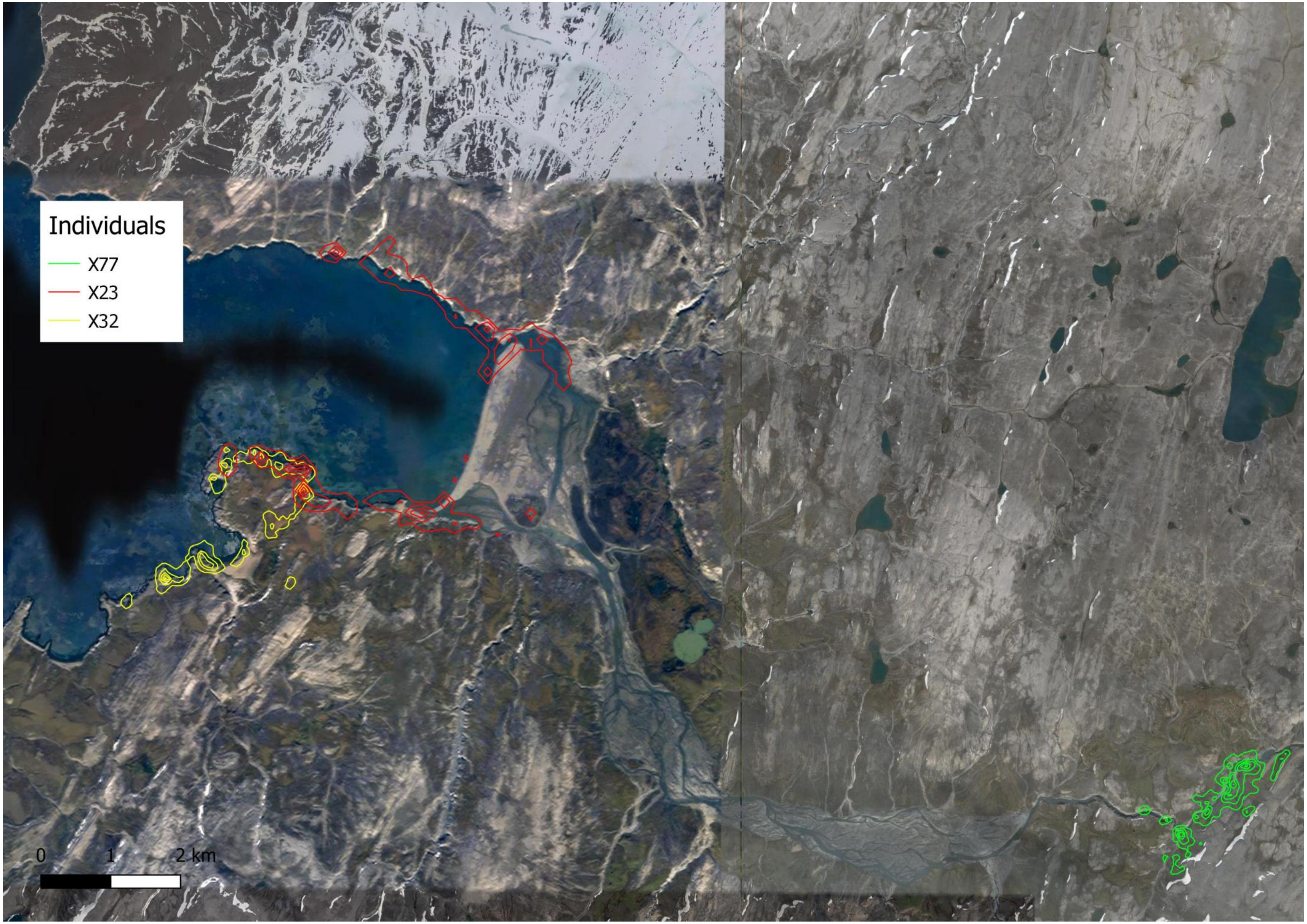
Individuals
X77
X23
X32
0
1
2 km

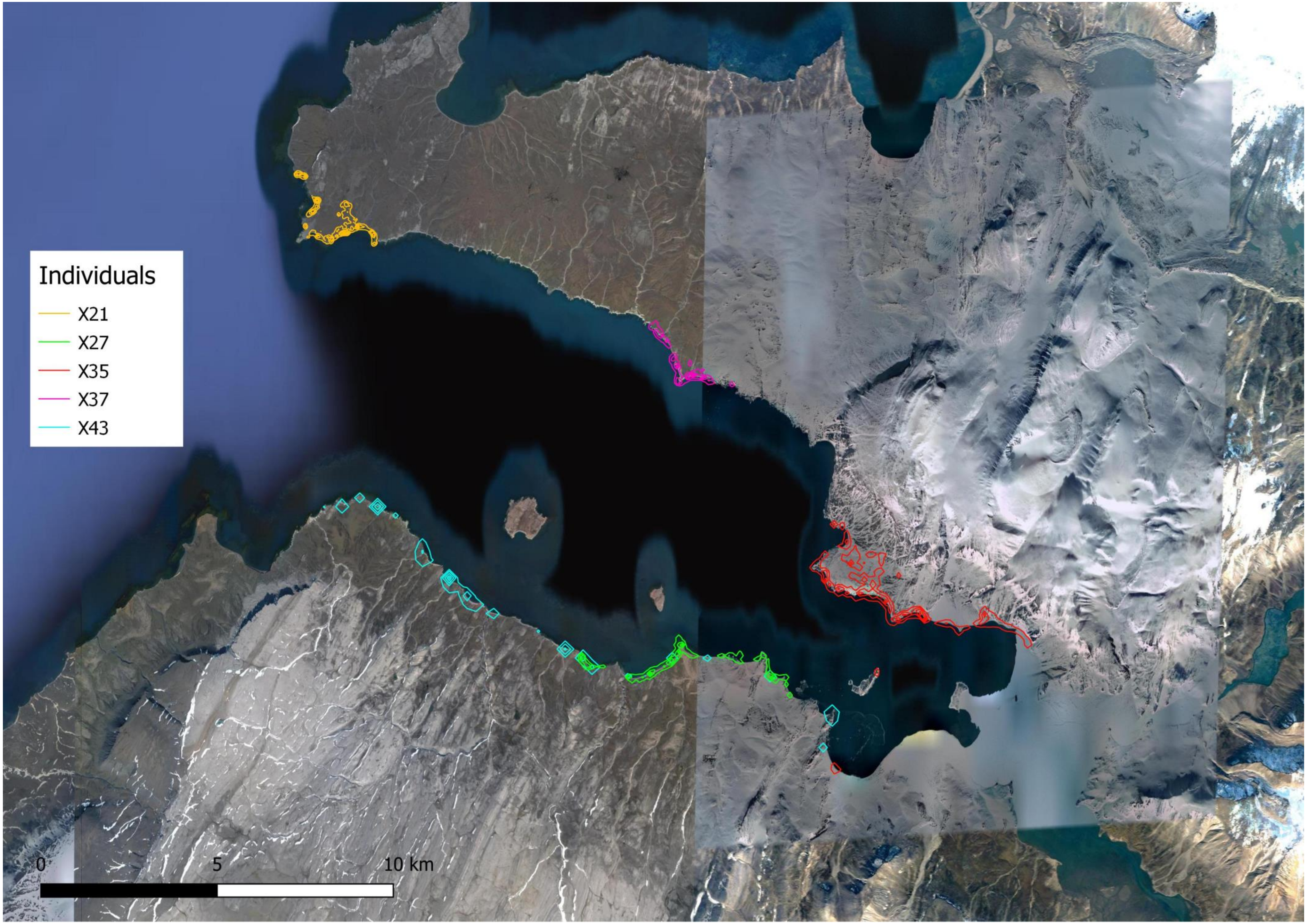
Individuals
X21
X27
X35
X37
X43
0
5
10 km

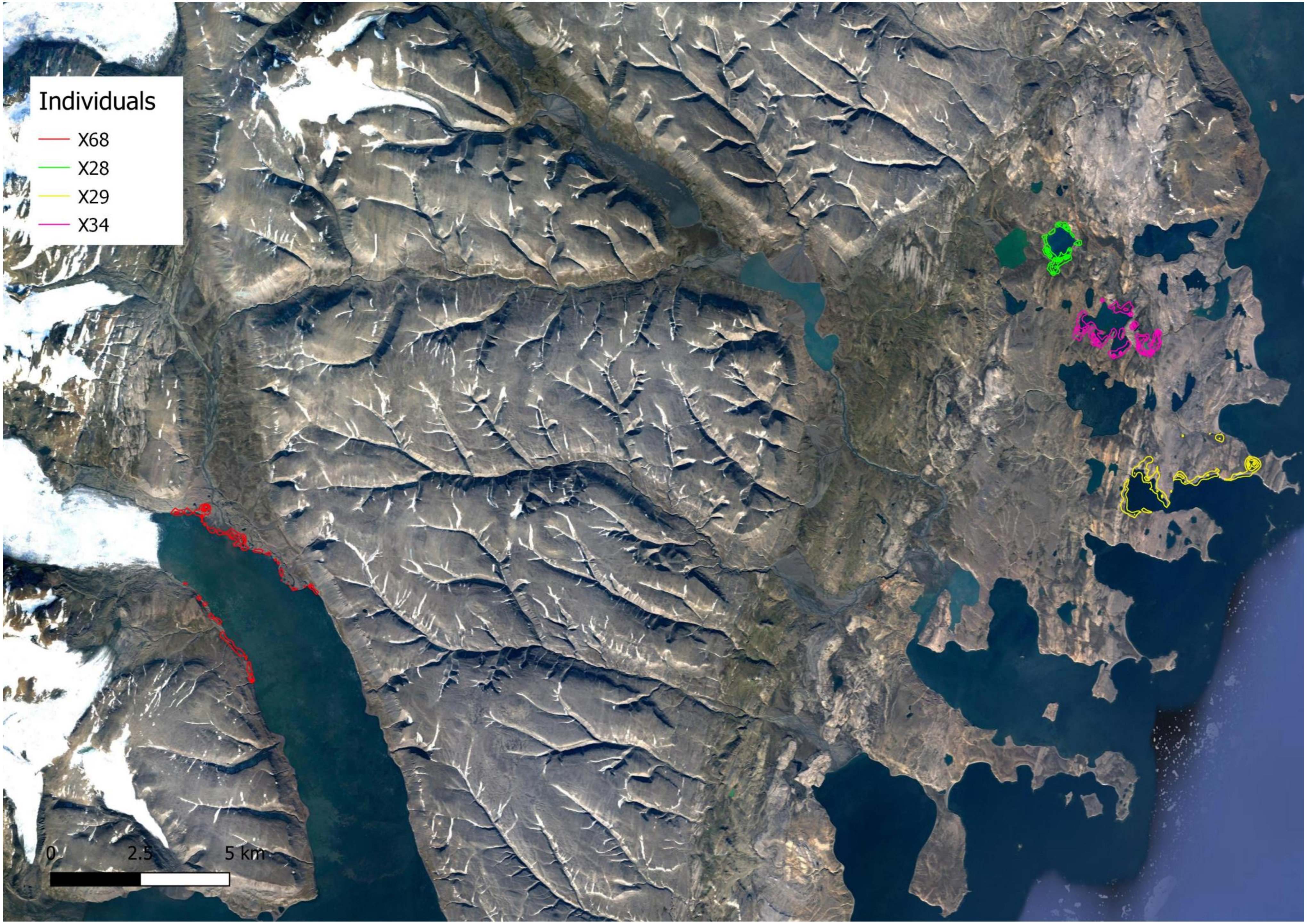
Individuals
X68
X28
X29
X34
0
2.5
5 km

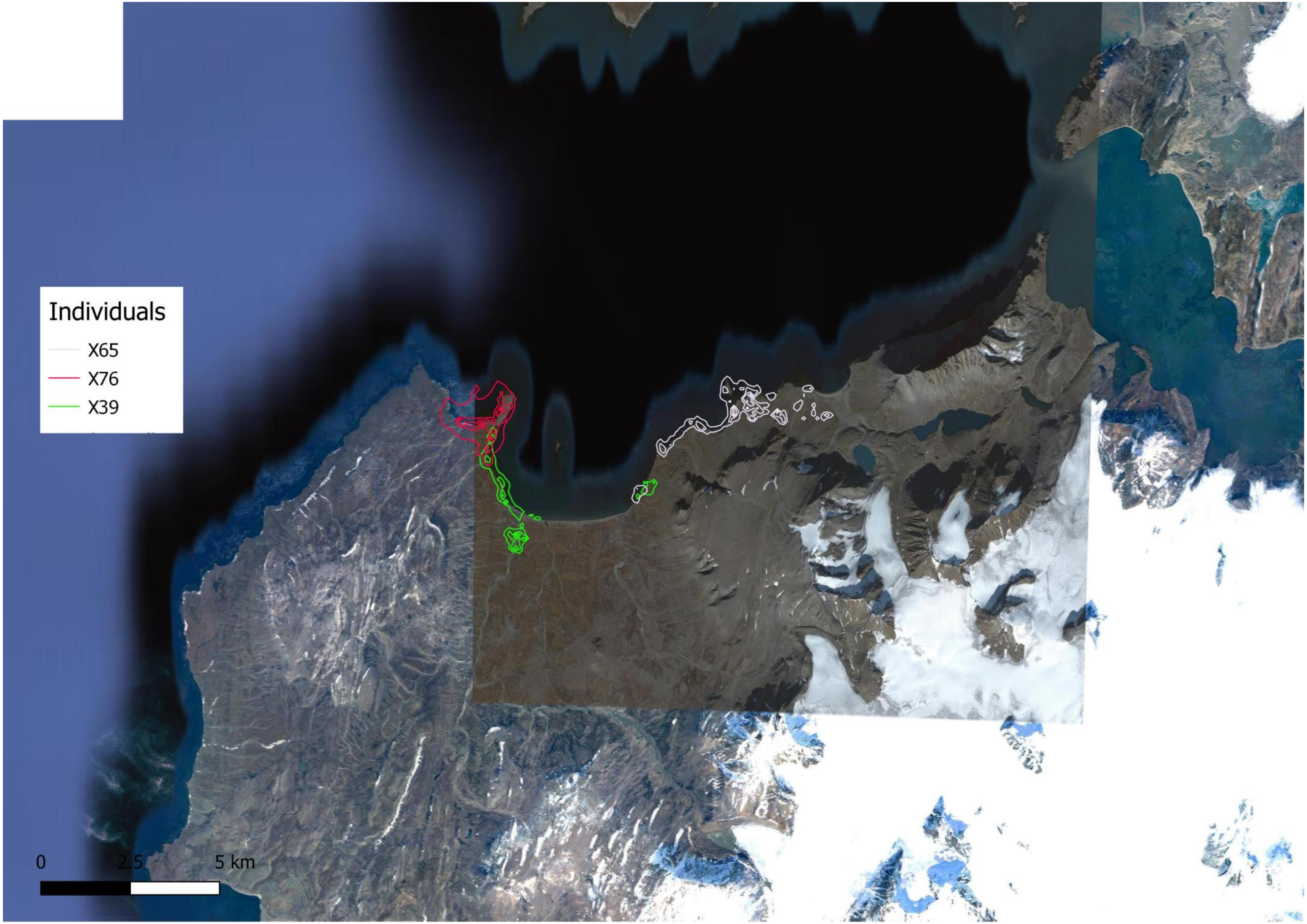
Individuals
X65
X76
X39
0
2.5
5 km

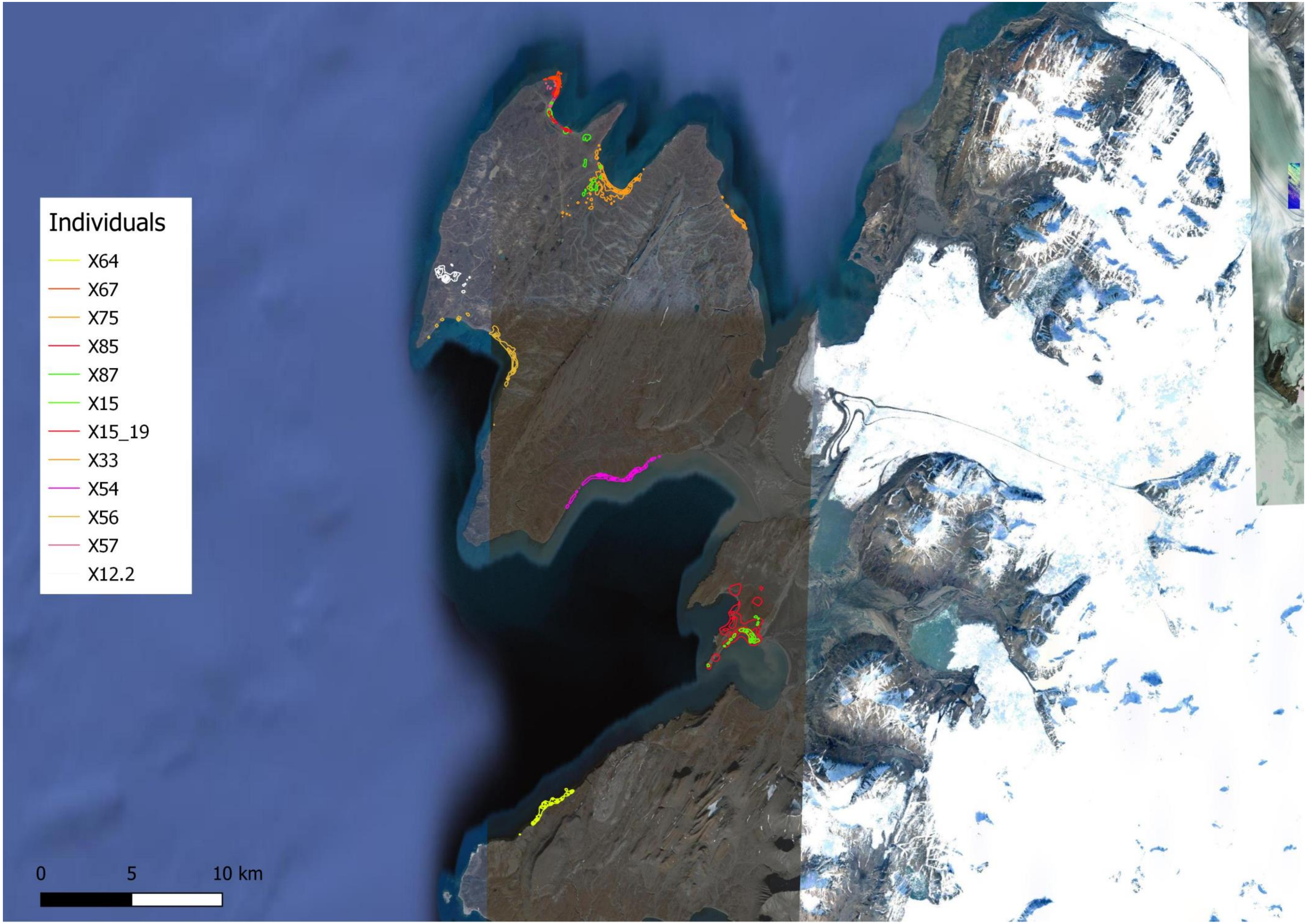
Individuals
X64
X67
X75
X85
X87
X15
X15_19
X33
X54
X56
X57
X12.2
0
5
10 km

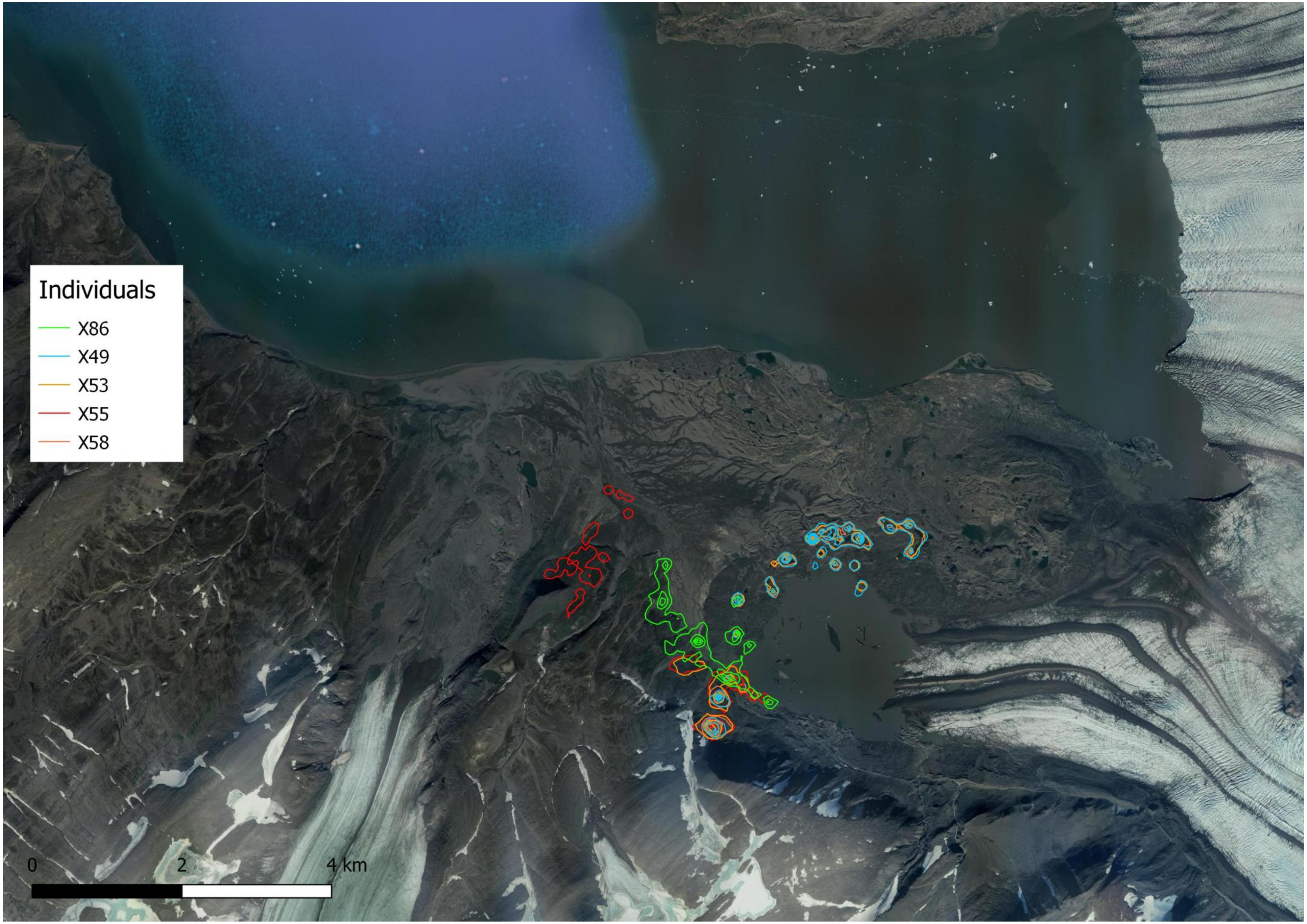
Individuals
X86
X49
X53
X55
X58
0
2
4 km

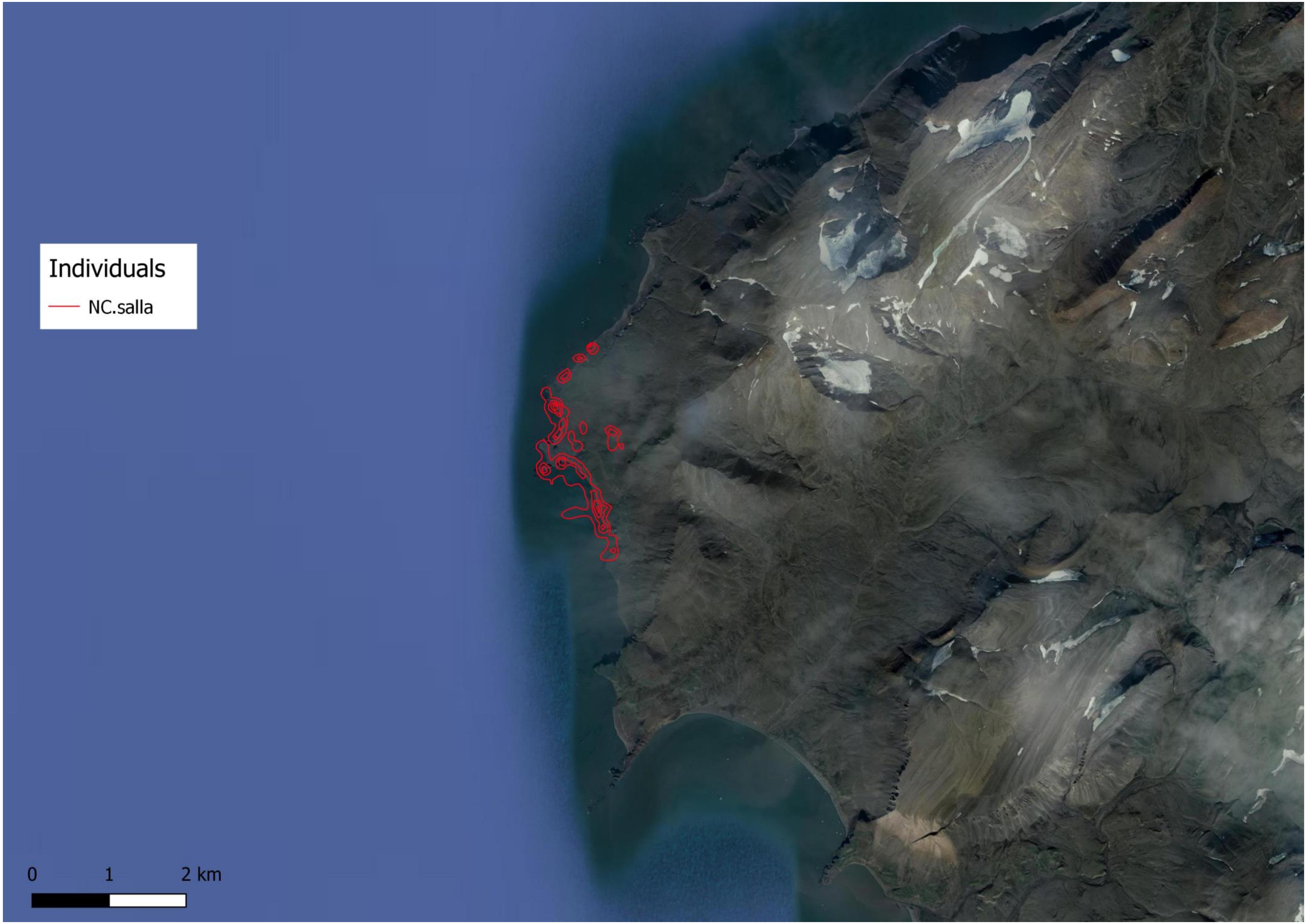
Individuals
NC.salla
0
1
2 km

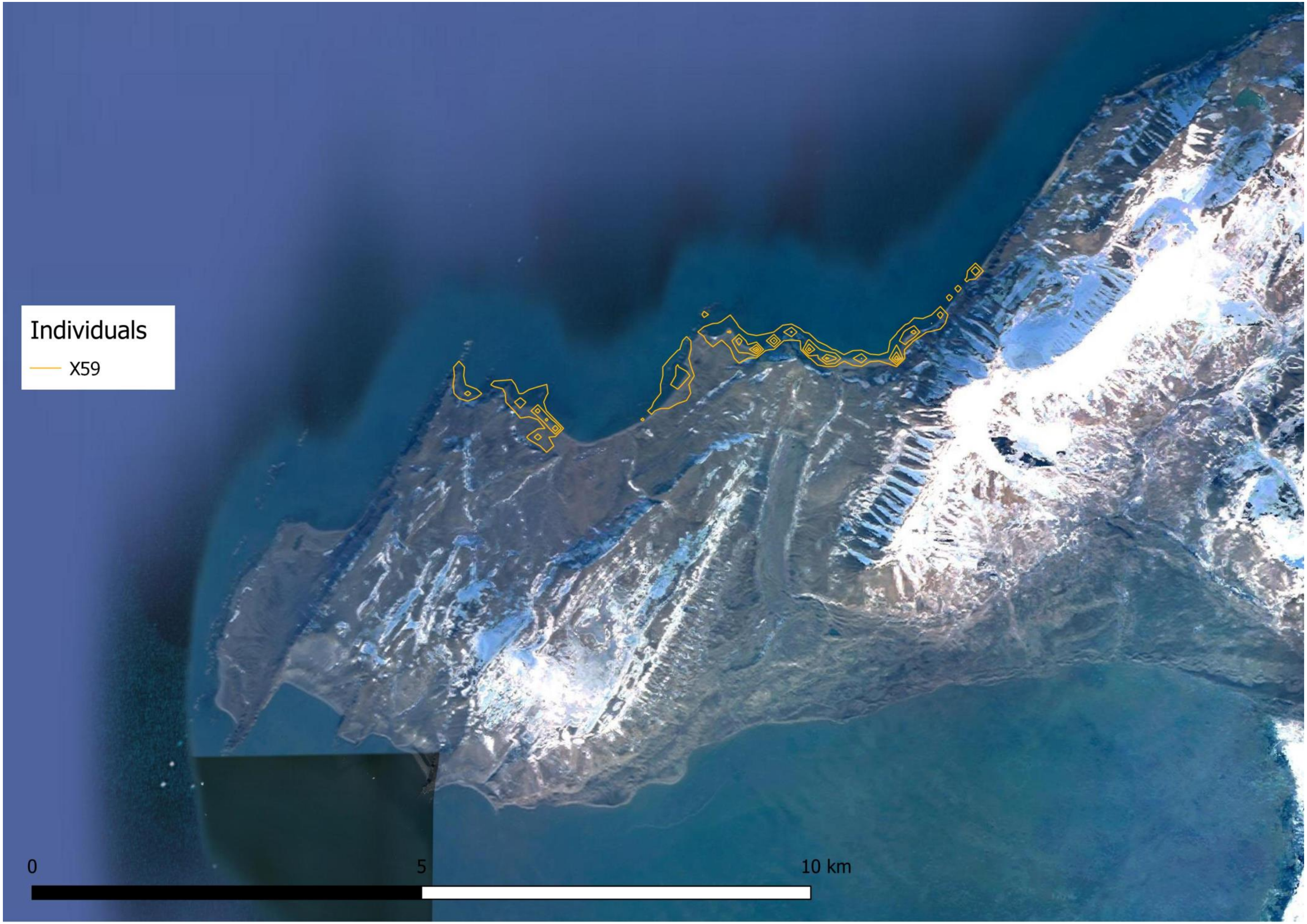

Individuals
X59
0
5
10 km

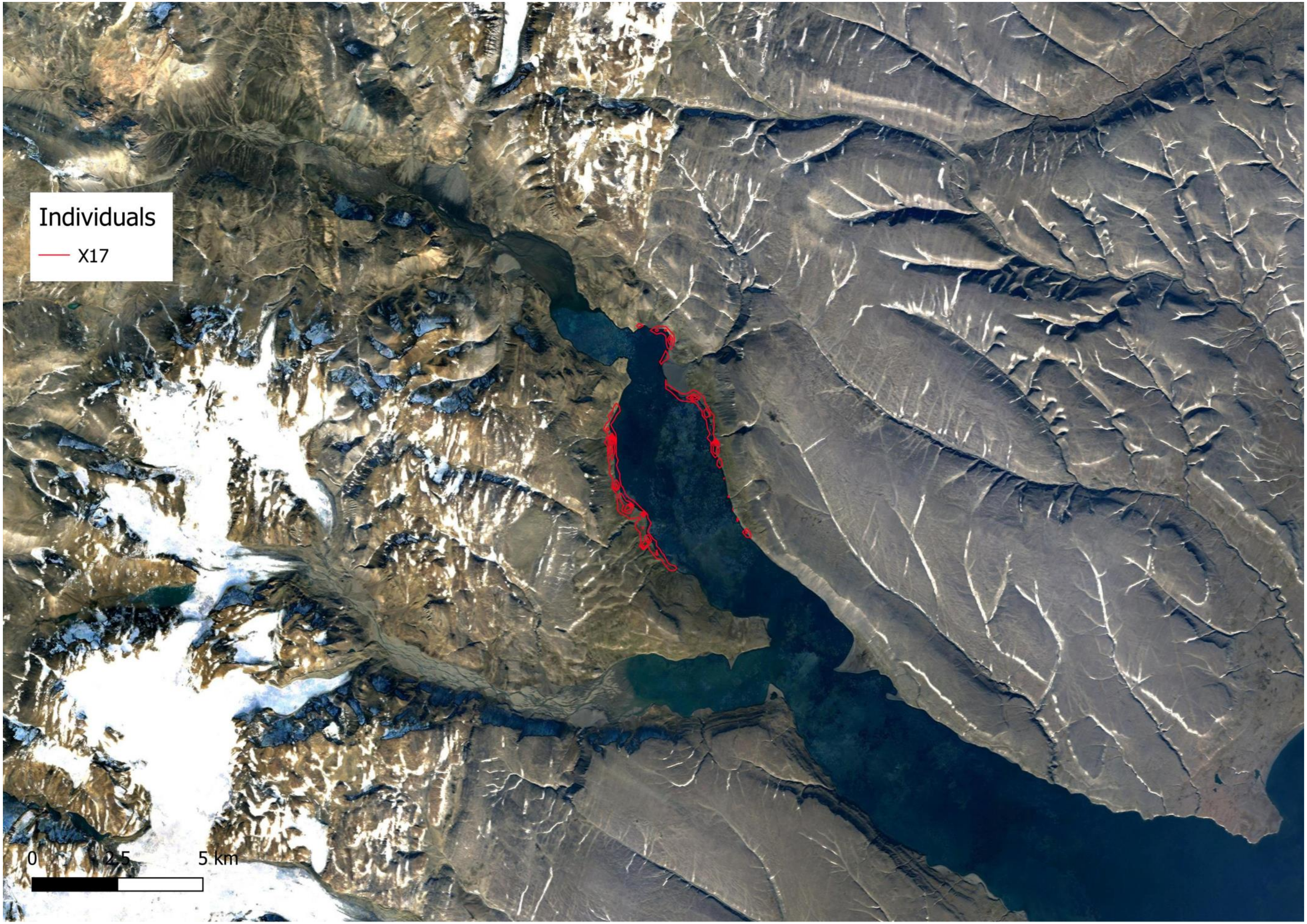
Individuals
X17
0
2.5
5 km

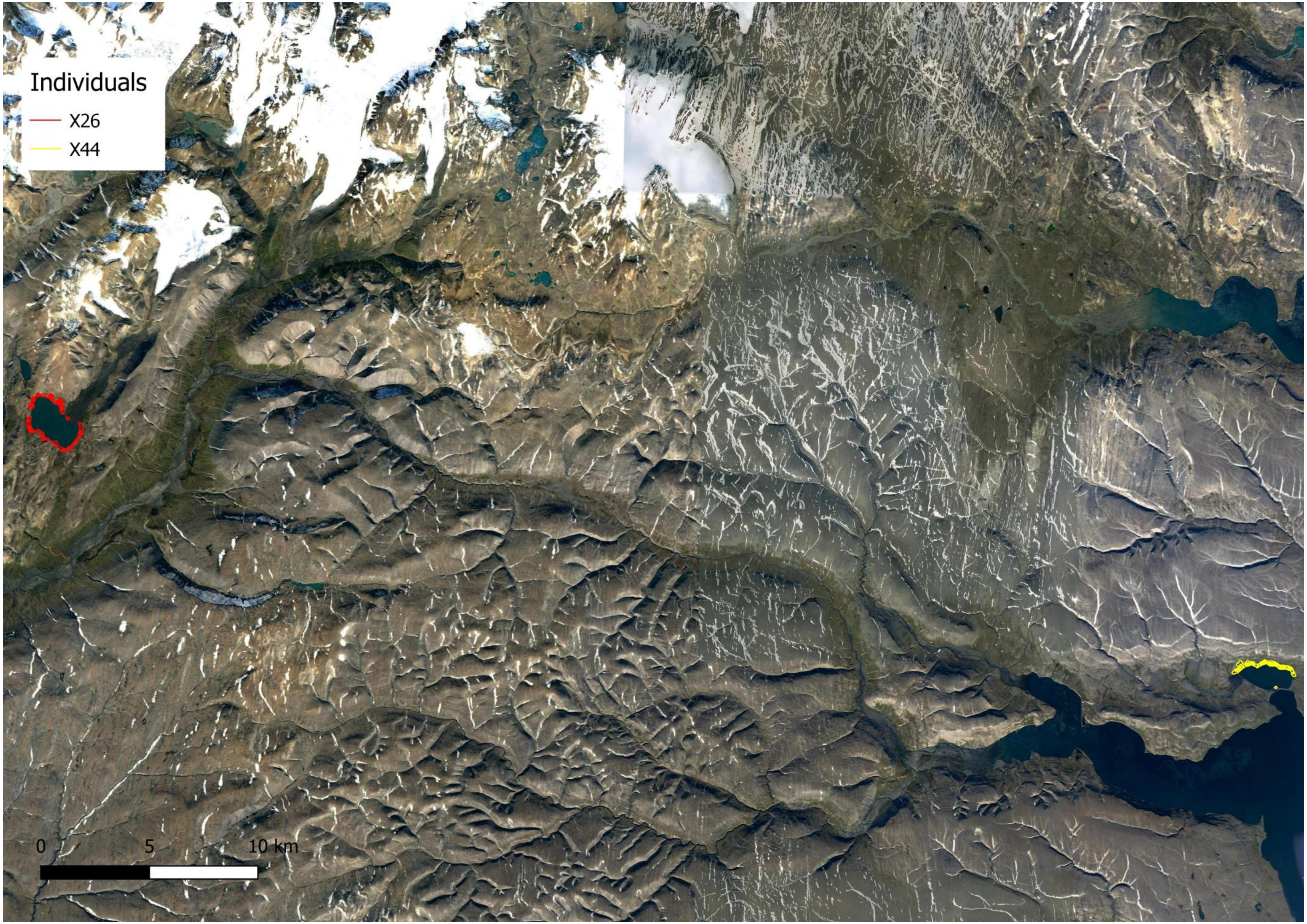

Individuals
X26
X44
0
5
10 km
