# Supplementary material for: Birds of three worlds: moult migration to high Arctic expands a boreal-temperate flyway to a third biome
Source: Mov Ecol. 2021 Sep 15;9:47. doi: 10.1186/s40462-021-00284-4 (PMC8444479; doi:10.1186/s40462-021-00284-4)
Supplement: Supplementary file 2 — Additional file 2. Table 1. Individual data on taiga bean geese marked with GPS-transmitters during 2019–2020. [file 40462_2021_284_MOESM2_ESM.pdf]

Table 1. Individual data on taiga bean geese marked with GPS-transmitters during 2019–2020.

Bird ID	Marking date (yyyy-mm-dd)	Marking site	Sex	Breeding status 2019	Breeding success 2019	Breeding status 2020	Breeding success 2020	Note
X32	2019-05-19	Salla	male	non-breeding		non-breeding		
X31	2019-05-06	Salla	female	non-breeding				No data after 2019/06/06
X29	2019-05-06	Salla	female	breeding	successful	non-breeding		
X28	2019-05-01	Lieksa	female	non-breeding		non-breeding		
X27	2019-05-06	Salla	female	non-breeding		non-breeding		
X26	2019-04-26	Pudasjärvi	female	non-breeding		breeding	unsuccessful	
X25 (1.)	2019-04-29	Lieksa	female	breeding				Bird died 2019/07/06
X25 (2.)	2019-07-17	Utajärvi	female	breeding				No data after 2019/10/02
X24	2019-04-26	Pudasjärvi	male	breeding	successful	non-breeding		
X23	2019-04-22	Virrat	female	breeding	unsuccessful	breeding	unsuccessful	
X22	2019-04-23	Virrat	male	breeding	unsuccessful	breeding		Bird died 2020/07/05
X21	2019-05-02	Salla	female	breeding	unsuccessful	non-breeding		
X17	2019-05-02	Salla	female	breeding	successful	breeding	unsuccessful	
X15	2019-04-28	Salla	female	non-breeding		non-breeding		
X14 (1.)	2019-04-27	Salla	female	breeding				Bird died 2019/07/01
X14 (2.)	2019-08-28	Salla	female	breeding	successful	non-breeding		
X13 (1.)	2019-04-24	Salla	female	breeding				Bird died 2019/06/07
X13 (2.)	2019-07-17	Utajärvi	female	breeding	successful	breeding	successful	
X12	2019-04-24	Salla	female	breeding	unsuccessful	non-breeding		
NC (Salla)	2019-05-02	Salla	male	breeding	successful	non-breeding		
NC (Ruunaa)	2019-05-01	Lieksa	female	non-breeding				No data after 2019/06/04
NC (Lieksa)	2019-05-01	Lieksa	male	non-breeding		breeding		Bird died 2020/06/07
X33	2020-04-08	Liperi	female			non-breeding		
X34	2020-04-28	Virrat	male			breeding	unsuccessful	
X35	2020-04-03	Outokumpu	female			breeding	unsuccessful	
X36	2020-04-03	Outokumpu	female			breeding		Bird died 2020/05/24
X37	2020-04-08	Liperi	female			breeding	unsuccessful	
X39	2020-04-08	Liperi	female			breeding	unsuccessful	
X42	2020-04-08	Liperi	female			breeding	successful	
X43	2020-04-08	Liperi	female			non-breeding		
X44	2020-04-16	Outokumpu	female			non-breeding		
X45	2020-04-03	Outokumpu	female			breeding	successful	
X46	2020-04-03	Outokumpu	female			breeding	successful	
X47	2020-04-28	Virrat	male			breeding	unsuccessful	
X48	2020-04-03	Outokumpu	female					No data after 2020/05/02
X49	2010-04-16	Outokumpu	female			non-breeding		
X52	2020-05-02	Lieksa	female			breeding	successful	
X53	2020-04-16	Outokumpu	male			non-breeding		
X54	2020-04-20	Virrat	female			breeding	unsuccessful	
X55	2020-04-16	Outokumpu	female			non-breeding		
X56	2020-04-20	Virrat	female			breeding	unsuccessful	
X57	2020-04-20	Virrat	female			breeding	unsuccessful	
X58	2020-04-16	Outokumpu	male			non-breeding		
X59	2020-05-02	Lieksa	female			breeding	unsuccessful	
X62	2020-04-23	Pudasjärvi	female			non-breeding		
X63	2020-04-08	Liperi	female			breeding	successful	
X64	2020-05-04	Salla	female			non-breeding		
X65	2020-05-16	Salla	female			non-breeding		
X67	2020-04-29	Pudasjärvi	female			non-breeding		
X68	2020-04-28	Pudasjärvi	female			breeding	unsuccessful	
X75	2020-05-05	Salla	female			non-breeding		
X76	2020-05-10	Salla	female			non-breeding		
X77	2020-05-07	Salla	female			non-breeding		
X85	2020-06-08	Salla	female			non-breeding		
X86	2020-05-16	Salla	female			non-breeding		
X87	2020-05-07	Salla	female			non-breeding		
NC (itkiin)	2020-05-04	Lieksa	female			non-breeding		
