# Supplementary material for: Birds of three worlds: moult migration to high Arctic expands a boreal-temperate flyway to a third biome
Source: Mov Ecol. 2021 Sep 15;9:47. doi: 10.1186/s40462-021-00284-4 (PMC8444479; doi:10.1186/s40462-021-00284-4)
Supplement: Supplementary file 3 — Additional file 3. Table 2. Length of migration routes, duration of migration and yearly additional journey caused by moult migration. [file 40462_2021_284_MOESM3_ESM.pdf]

Table 2. Explanation for the sample sizes used in the analysis. Total number of birds with GPS transmitters in the spring 2019 was 19 and three more transmitters were deployed during the summer. In the spring 2020, 50 birds were fitted with GPS-transmitters and transmitters were not deployed during the summer.

**Event**	**n**	**Explanation for sample size**
Beginning of moult migration (non-breeders) in 2019	7	All (19) - breeders (11) - one that stopped sending before departure
Beginning of moult migration (non-breeders) in 2020	26	All (50) - 22 breeders - two birds from pairs
Breeding success 2019	10	All breeders (11) - one dead (faith of brood unknown)
Breeding success 2020	20	All breeders (22) - two dead (faith of brood unknown)
Time lag between nest/brood loss and the beginning of moult migration	16	Failed breeders in 2019 (4) and 2020 (14) - one predated during incubation - one to avoid pseudorep.
Arrival to NZ in 2019	10	All (19) - dead (3) - stopped sending (2) - successful breeders (4)
Arrival to NZ in 2020	39	All (50) - successful breeders (6) - dead (3) - two birds from pairs
Time lag between arrival to NZ and beginning of moult	42	All that arrived to NZ in 2019 (10) and 2020 (41) - seven to avoid pseudorep. - two birds from pairs
Beginning and end of moult in 2019	10	All that arrived to NZ in 2019
Beginning and end of moult in 2020	41	All that arrived to NZ in 2020
Beginning and end of moult in merged data	44	All that arrived to NZ in 2019 (10) and 2020 (41) - seven to avoid pseudorep. - two birds from pairs
Utilization distribution during moult	41	All that arrived to NZ in 2019 (10) and 2020 (41) - seven to avoid pseudoreplication - three with location intreval of 2 h
Departure from NZ 2019	10	All that arrived to NZ in 2019
Departure from NZ 2020	39	All that arrived to NZ in 2020 - two birds from pairs
Time lag between the end of moult and departure from NZ in 2019	10	All that arrived to NZ in 2019
Time lag between the end of moult and departure from NZ in 2020	39	All that arrived to NZ in 2020 - two birds from pairs
Arrival to Sweden 2019 (moult migrants)	10	All that arrived to NZ in 2019
Arrival of to Sweden 2020 (moult migrants)	38	All that arrived to NZ in 2020 - two birds from pairs - one died in Finland
Arrival of to Sweden in 2019 (successful breeders)	7	Successful breeders (4) + three birds marked during summer in Finland (all successful breeders)
Arrival of to Sweden in 2020 (successful breeders)	6	Successful breeders
Stopovers in Finland 2019	10	All that arrived to NZ in 2019
Stopovers in Finland 2020	39	All that arrived to NZ in 2020 - two birds from pairs
