# Supplementary material for: Birds of three worlds: moult migration to high Arctic expands a boreal-temperate flyway to a third biome
Source: Mov Ecol. 2021 Sep 15;9:47. doi: 10.1186/s40462-021-00284-4 (PMC8444479; doi:10.1186/s40462-021-00284-4)
Supplement: Supplementary file 4 — Additional file 4. Table 3. Explanation of sample sizes used in the analysis. [file 40462_2021_284_MOESM4_ESM.pdf]

Table 3. Length of migration routes (i.e. distance between starting point and endpoint), duration of migrations and yearly additional journey (moult migration distance + autumn migration distance - distance between breeding and wintering sites) caused by moult migration for satellite tracked taiga bean geese in 2019 and 2020.

Track	Marking site	Breeding status	Moult mig. length (km)	Autumn mig. length (km)	Moult mig. duration (days)	Autumn mig. duration (days)	Additional journey (km)	Annual mig. distance (km)
Moult migrants								
NC.itkiin-2020	Lieksa	non-breeding	2810	4745	1.85	15.76	5603	9506
NC.lieksa-2019	Lieksa	non-breeding	2971	4904	7.61	3.69	5924	9827
NC.salla-2020	Salla	non-breeding	3280	5087	5.24	10.89	6411	10323
X12.1-2019	Salla	breeding	3082	4899	1.5	1.46	6025	9937
X12.2-2020	Salla	non-breeding	3088	4905	1.72	16.33	6036	9948
X14.2-2020	Salla	non-breeding	3014	4819	3.71	16.74	5877	9789
X15-2019	Salla	non-breeding	3144	4954	3.13	17.65	6143	10055
X15-2020	Salla	non-breeding	3144	4954	1.85	55.38	6143	10055
X17-2020	Salla	breeding	3165	4957	0.83	6.48	6166	10078
X21-2019	Salla	breeding	3129	4942	1.79	1.25	6115	10027
X21-2020	Salla	non-breeding	2998	4808	1.27	12.73	5851	9763
X22-2019	Virrat	breeding	3796	4936	2.62	9.06	7521	9942
X23-2019	Virrat	breeding	3628	4768	3.4	1.88	7186	9607
X23-2020	Virrat	breeding	3628	4768	1.1	7.28	7186	9607
X24-2020	Pudasjärvi	non-breeding	3229	4813	3.71	34.6	6339	9743
X26-2019	Pudasjärvi	non-breeding	3095	4677	13.17	2.84	6069	9473
X26-2020	Pudasjärvi	breeding	3095	4677	1.56	17.54	6069	9473
X27-2019	Salla	non-breeding	3034	4841	17.24	1.87	5918	9830
X27-2020	Salla	non-breeding	3034	4841	0.77	37.56	5918	9830
X28-2019	Lieksa	non-breeding	2908	4842	1.56	2.29	5799	9701
X28-2020	Lieksa	non-breeding	3227	5149	3.35	3.19	6424	10327
X29-2020	Salla	non-breeding	3383	5167	16.56	14.32	6594	10506
X32-2019	Salla	non-breeding	2927	4740	3.43	2.19	5711	9623
X32-2020	Salla	non-breeding	2883	4695	1.09	49.14	5622	9534
X33-2020	Liperi	non-breeding	2505	4930	4.67	1.93	5001	9870
X34-2020	Virrat	breeding	4025	5151	1.21	12	7965	10387
X35-2020	Outokumpu	breeding	2460	4863	1.03	18.59	4905	9740
X37-2020	Liperi	breeding	2811	4844	1.19	11.44	5615	9695
X39-2020	Liperi	breeding	2548	4900	6.22	3.21	5068	9828
X43-2020	Liperi	non-breeding	2237	4792	1.99	26.39	4397	9660
X44-2020	Outokumpu	non-breeding	2919	4844	1.42	34.43	5811	9714
X47-2020	Virrat	breeding	3515	4659	1.25	2.94	6963	9384
X49-2020	Outokumpu	non-breeding	2782	5105	0.87	4.04	5443	10331
X53-2020	Outokumpu	non-breeding	2780	5103	1.89	4.09	5439	10326
X54-2020	NA	NA	3971	5105	NA	NA	7865	10286
X55-2020	Outokumpu	non-breeding	2575	4910	1.16	3.12	5041	9929
X56-2020	Virrat	breeding	3782	4924	2.68	3.1	7495	9916
X57-2020	Virrat	breeding	3966	5101	2.59	9.13	7856	10277
X58-2020	Outokumpu	non-breeding	2834	5158	1.16	3.71	5549	10436
X59-2020	Lieksa	breeding	2883	4815	3.73	9.34	5746	9649
X62-2020	Pudasjärvi	non-breeding	3330	4918	3.72	11.45	6546	9950
X64-2020	Salla	non-breeding	3114	4923	3.47	27.49	6082	9994
X65-2020	Salla	non-breeding	3107	4923	1.17	34	6074	9986
X67-2020	Pudasjärvi	non-breeding	3468	5041	4.54	11.62	6807	10211
X68-2020	Pudasjärvi	breeding	3366	4955	1.12	3.7	6620	10023
X75-2020	Salla	non-breeding	3085	4896	1.44	11.58	6025	9937
X76-2020	Salla	non-breeding	2997	4805	7.5	57.58	5846	9759
X77-2020	Salla	non-breeding	3107	4923	2.12	27.64	6075	9987
X85-2020	Salla	non-breeding	3295	5099	0.51	27.52	6438	10350
X86-2020	Salla	non-breeding	3109	4924	1.32	10.38	6077	9989
X87-2020	Salla	non-breeding	2932	4745	2.03	26.62	5721	9633
Successful breeders (no moult migration)								
NC.salla-2019	Salla	breeding	NA	1956	NA	3.08	NA	3912
X13.2-2019	Utajärvi	breeding	NA	1689	NA	0.46	NA	3379
X13.2-2020	Utajärvi	breeding	NA	1689	NA	36.24	NA	3379
X14.2-2019	Salla	breeding	NA	1956	NA	4.15	NA	3912
X17-2019	Salla	breeding	NA	1956	NA	3.03	NA	3912
X24-2019	Pudasjärvi	breeding	NA	1702	NA	1.12	NA	3404
X29-2019	Salla	breeding	NA	1956	NA	39.83	NA	3912
X42-2020	Liperi	breeding	NA	2047	NA	21.38	NA	4093
X45-2020	Outokumpu	breeding	NA	3998	NA	50.35	NA	7996
X46-2020	Outokumpu	breeding	NA	2853	NA	3.9	NA	5705
X52-2020	Lieksa	breeding	NA	1951	NA	0.49	NA	3903
X63-2020	Liperi	breeding	NA	3303	NA	62.45	NA	6605
